# Spatial Isolation of Single Copper(I) Sites for Cascade Enzyme‐Like Catalysis and Simultaneous Ferroptosis/Cuproptosis Boosted Immunotherapy

**DOI:** 10.1002/EXP.20240275

**Published:** 2025-03-06

**Authors:** Yuanyuan Zhang, Shengnan Ya, Jingnan Huang, Yangyang Ju, Xueyang Fang, Xinteng Ouyang, Qingdong Zeng, Xinyao Zhou, Xiyun Yan, Guohui Nie, Kelong Fan, Bin Zhang

**Affiliations:** ^1^ Department of Otolaryngology Shenzhen Key Laboratory of Nanozymes and Translational Cancer Research Shenzhen Institute of Translational Medicine The First Affiliated Hospital of Shenzhen University Shenzhen Second People's Hospital Shenzhen University Medical School Shenzhen China; ^2^ School of Medical Imageology Wannan Medical College Wuhu China; ^3^ Department of Gastroenterology The First Affiliated Hospital (Shenzhen People's Hospital) Southern University of Science and Technology Shenzhen China; ^4^ Fischell Department of Bioengineering University of Maryland College Park Maryland USA; ^5^ CAS Engineering Laboratory for Nanozyme Key Laboratory of Biomacromolecules (CAS) CAS Center for Excellence in Biomacromolecules Institute of Biophysics Chinese Academy of Sciences Beijing China; ^6^ Nanozyme Laboratory in Zhongyuan Henan Academy of Innovations in Medical Science Zhengzhou Henan China

**Keywords:** cuproptosis, ferroptosis, immunogenic cell death, photothermal‐amplified ROS storms, single‐site nanozyme

## Abstract

Nanozyme‐based immunogenic cell death (ICD) inducers that effectively induce a strong immune response via enzyme‐like process have attracted great attention, but how to ensure controllable active sites and maximize site utilization remains a problem. Here, we report a structurally well‐defined and highly functional single‐site copper(I) nanomodulators termed CuNTD, constructed by precisely anchoring atomically dispersed self‐assembly S‐Cu(I)‐S sites onto a two‐dimensional Ti_3_C_2_ surface. Leveraging Cu^+^ with a higher catalytic efficiency than Cu^2+^, CuNTD generates reactive oxygen species (ROS) storms through photothermal‐enhanced cascade catalysis, further inducing mitochondrial dysfunction, ferroptosis and cuproptosis. Multifunctional CuNTD triggers strong ICD through cascade‐regulatory pathways of photothermal‐amplified ROS storms, cuproptosis and ferroptosis, effectively promoting dendritic cell maturation while reducing monotherapies side effects and resistance. In vivo, CuNTD combined with FDA‐approved immunoadjuvants significantly prolong the survival of mice. With its demonstrated biosafety and high efficiency as an ICD inducer, this study provides a promising framework for advancing augmented tumor immunotherapy with significant clinical potential.

## Graphical abstract

Self‐assembled S‐Cu(I)‐S sites were spatially isolated onto a two‐dimensional Ti_3_C_2_ surface to produce a structurally well‐defined and highly functional single‐site copper(I) nanomodulator CuNTD, which can induce severe immunogenic cell death through multiple regulatory pathways of synergistic cascade photothermal‐amplified ROS storms, cuproptosis and ferroptosis for cancer immunotherapy.

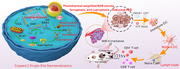

## 1 Introduction

Immunotherapy has achieved remarkable success in treating cancer, particularly for triple‐negative breast cancer (TNBC), which is characterized by a high rate of relapse and metastases, [1]. As one of the most promising therapeutic approaches, immunotherapy provides the potential for long‐term immune activation and complete tumor eradication. However, TNBC is classified as an immunologically cold tumor with a limited response to immunotherapy due to immunosuppression of the tumor microenvironment (TME)[2]. Previous findings demonstrate that immunogenic cell death (ICD) could induce the release of damage‐associated molecular pattern signals (DAMPs) and tumor‐associated antigens (TAAs), and facilitate the maturation of dendritic cells (DCs) and the infiltration of CD8^+^ T lymphocytes [3]. This process reverses the immune “cold” into a “hot” inflammatory tumor and triggers a durable anti‐tumor immune response [4]. Although ICD can be induced by chemotherapy, radiotherapy, and phototherapy [5], achieving effective and safe induction of ICD remains a significant challenge in clinical practice.

Reactive oxygen species (ROS) production plays a pivotal role in inducing ICD [6]. Recently, ROS‐mediated catalytic therapies utilizing nanozymes have emerged as promising cancer treatments. Copper, as a crucial trace element [7], is a cofactor in redox reactions required for many enzymes and is extensively engaged in in vivo redox processes [8]. Copper‐based nanomaterials, in previous studies, can simulate natural enzymes like catalase (CAT), glutathione peroxidase (GPx), peroxidase (POD), and oxidase (OXD) [9]. These enzymes specifically catalyze excess H_2_O_2_ and deplete glutathione (GSH), producing highly toxic ROS that efficiently kill tumor cells [10]. Additionally, cuproptosis, a newly discovered copper‐reliant type of programmed cell death that differs significantly from apoptosis, necrosis, autophagy, and ferroptosis, shows enormous potential in addressing the shortcomings of existing cancer therapies [11]. This process is characterized by the excessive intracellular copper‐triggered lipoylated dihydrolipoamide S‐acetyltransferase (DLAT) agglomeration and Fe‐S protein depletion, the occurrence of which might lead to proteotoxic stress and eventual cell death [12]. Compared to iron, copper exhibits higher Fenton catalytic efficiency, triggering ferroptosis by deactivating glutathione peroxidase 4 (GPX4) and promoting the peroxidation process of lethal lipids [13]. Although copper‐based nanoplatform can synergistically induce ICD by triggering various therapeutic modalities, its higher toxicity than iron and manganese has hindered its clinical application [14].

To address these challenges, single atom nanozymes (SAzymes) garnered attention in catalytic therapy due to their well‐defined active sites, high atomic utilization, admiring catalytic activity and controllable selectivity [15]. SAzymes confine, anchor, or coordinate dispersed metal atoms to form well‐defined structures and uniform active sites [16]. Based on the efficient catalytic performance of copper and the discovery of cuproptosis, the application of single‐atom copper enzymes in biomedicine has received extensive attention [17]. Single‐atom copper enzymes maximize the catalytic performance of copper to enhance their therapeutic efficiency [18], but the balance between the therapeutic efficiency and biocompatibility of metal nanozymes remains a challenge. Compared to traditional nanozymes, SAzymes have higher atomic utilization, but most reported SAzymes' active sites are still primarily restricted to surface atoms, and their internal metal atoms are underutilized. Two‐dimensional (2D) nanomaterials, with their planar topology and ultrathin thickness, have a high specific surface area, making them a powerful platform for supporting atomically dispersed metal species. Among them, 2D Ti_3_C_2_ is particularly effective due to its expansive specific surface area, modifiable hydrophilic surface, efficient photothermal conversion, and strong biocompatibility [19]. Furthermore, the photothermal effect can not only promote the catalytic activity of SAzymes but also facilitate ICD induction. Therefore, a 2D Ti_3_C_2_‐supported Cu SAzyme as nanomodulators was developed for photothermal‐amplified ROS storm, cuproptosis and ferroptosis cascade‐triggering ICD [20] to maximize the therapeutic properties of copper with minimal side effects.

**SCHEME 1:**
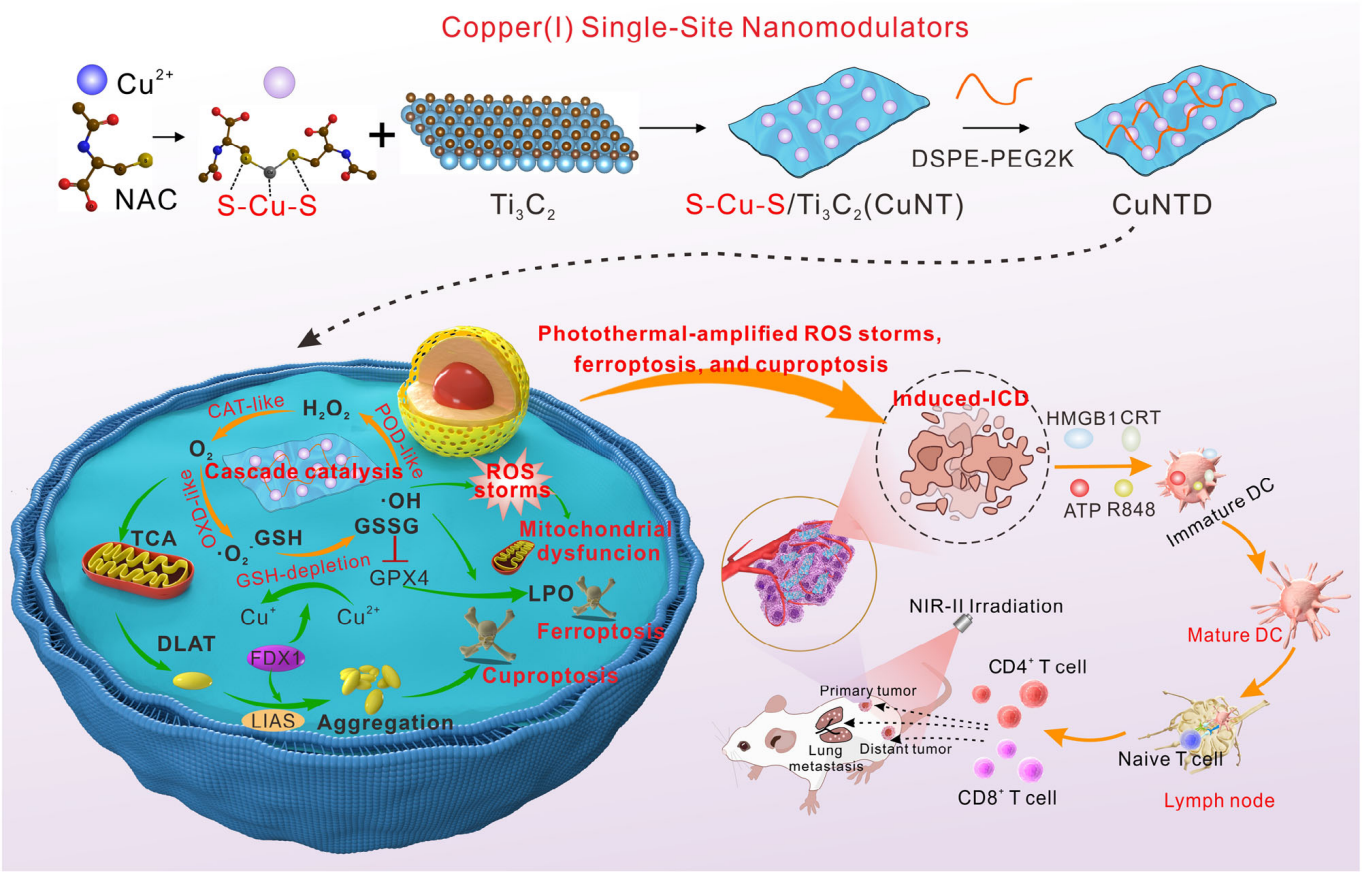
Schematic description of the synthesis process of CuNTD and the photothermal‐amplified ROS storms, ferroptosis, and cuproptosis, cascade‐inducing ICD augmented TNBC immunotherapy.

In this study, we integrated the biocompatible thiolate ligand *N*‐acetyl‐L‐cysteine (NAC) with copper ions to form S‐Cu(I)‐S through the strong Cu‐S coordination and the redox reaction. These isolated S‐Cu(I)‐S sites were self‐assembled onto the surface of 2D Ti_3_C_2_, resulting in the construction of a structurally well‐defined and highly functional Cu SAzyme termed CuNTD. Cu in CuNTD stays as Cu^+^, which exhibits a higher catalytic efficiency than Cu^2+^ and is vital for cuproptosis. Under photothermal stimulation, CuNTD demonstrated cascade catalytic activity, driving the catalytic H_2_O_2_ conversion as well as GSH depletion. This process produced ROS storms, causing mitochondrial dysfunction, lipid peroxidation‐mediated ferroptosis and stimulus‐responsive release of Cu, further inducing DLAT aggregation‐mediated cuproptosis. These mechanisms collectively triggered cells to release damage‐related molecular patterns (adenosine triphosphate (ATP), calreticulin (CRT), and high mobility group box 1 (HMGB1)), boosting ICD [21]. Based on the multifunctionality of CuNTD, we implemented a cascade‐augmented therapeutic efficacy that not only effectively ablated the tumor in situ but also strongly activated the ICD‐mediated immune response at the tumor site. In vivo, a single in situ injection of CuNTD with FDA‐approved immunoadjuvant such as R848 [22], which acts as a synthetic agonist for Toll‐like receptors 7 and 8 (TLR7/8) to effectively promote the aggregation of DC cells into mouse lymph nodes and induce the production of CD4^+^ and CD8^+^ effector T cells, provided the formation of durable immune memory and prolonged survival of mice. (Scheme 1)

## 2 Results and Discussion

### 2.1 Synthesis and Characterizations

**FIGURE 1:**
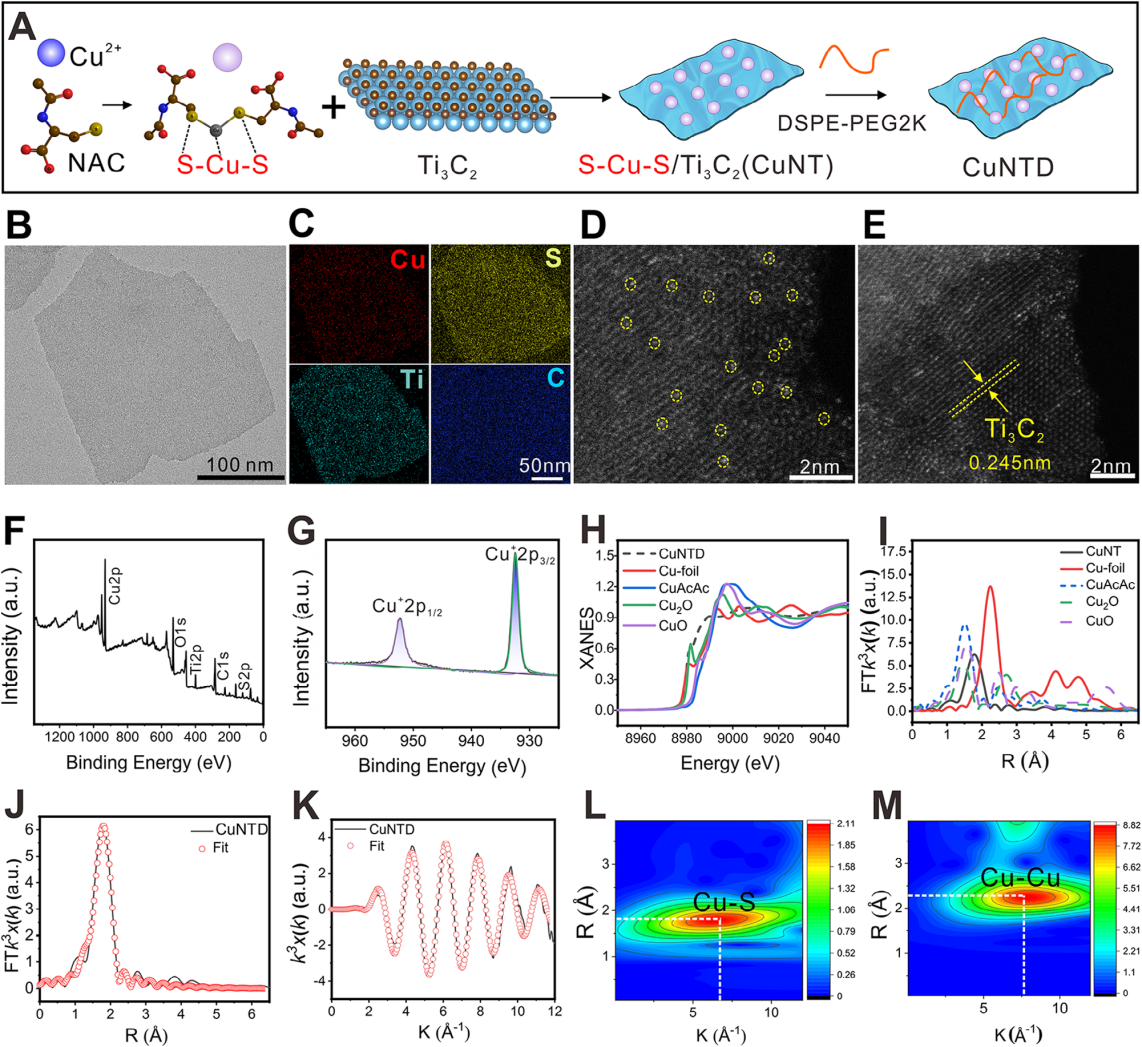
Preparation and characterizations of CuNTD. (A) A diagram showing the fabrication process of CuNTD. (B) TEM image of single layer CuNTD. (C) EDX mappings of Cu, S, Ti, and C in CuNTD. (D,E) HAADF‐STEM images of CuNTD. Individual Cu atoms are indicated by the yellow circles. (F,G) XPS analysis spectrum of CuNTD and high‐resolution profile of Cu2p. (H) XANES spectra at Cu K‐edge of Cu foil, Cu_2_O, CuO, CuAcAc, and CuNTD. (I) EXAFS spectra of Cu foil, Cu_2_O, CuO, CuAcAc, and CuNTD were Fourier transformed in the *R* space. (J) EXAFS fitting result of CuNTD at *R* space. (K) EXAFS fitting result of CuNTD at *k* space. (L,M) Wavelet transform of CuNTD and Cu foil.

The coordination‐driven self‐assembly method offers a versatile approach to functionalizing nanomaterials [15b]. Copper ions, known for their high coordination ability with sulfhydryl ligands, were paired with *N*‐acetylcysteine (NAC), [23] renowned for its metal chelating capability, resulting in the synthesis of Cu single‐atom nanomodulators (Figure 1A). Initially, atomically dispersed S‐Cu(I)‐S sites were formed by chelating NAC with Cu^2+^ (Figure S1), followed by the coordination‐driven self‐assembly between S‐Cu(I)‐S sites and Ti_3_C_2_ to produce CuNT. As shown in Figure S1A, the obvious loss of the ─S─H vibration stretching peak (2250 and 942 cm^−1^) associated with NAC was attributed to the successful formation of the Cu‐NAC complex. The formation of S─Cu─S was also proved by XPS (Figure S1B) and the Cu2p XPS spectrum revealed prominent peaks at 932.4 and 952.4 eV, which were assigned to the +1‐chemical valence of S─Cu─S and not the +2‐chemical valence of copper acetate. To enhance the biocompatibility of CuNT, amphiphilic DSPE‐PEG2000 was surface‐modified on CuNTD through hydrophobic interaction. It has been reported that Cu^+^ exhibits higher activity in Fenton‐like reaction compared to Cu^2+^, thereby enhancing •OH yield [24]. NAC possesses advantages for coordinating with metal with thiol ligand, regulating electronic environments, as well as a reducing agent. These benefits guarantee the +1 charge of Cu species against oxidation in TME and enhance the Cu loading, preventing them from aggregating or migrating into nanoparticles, ultimately boosting catalytic performance [15b]. Transmission electron microscopy (TEM) image revealed that CuNTD exhibits a single‐layer nanosheet with a size of ≈200 nm (Figure 1B), consistent with the dynamic light scattering (DLS) results in Figure S2. Further, the zeta potential of this surface‐modified DSPE‐PEG2000nanoplatform was measured to be −26 mV.

Further characterization using atomic force microscopy (AFM) indicated a thickness of approximately 2 nm for CuNTD (Figure S3A,B). Energy dispersive X‐ray spectroscopy (EDX) mapping (Figure 1C and Figure S4) demonstrated that Cu (red), S (yellow), Ti (green), and C (blue) elements are distributed homogeneously in CuNTD. Inductively coupled plasma optical emission spectrometry (ICP‐OES) was employed for determining the accurate metal loading, and the weight percentage of Cu is about 1.5 wt%, which is a high value among single‐atom catalysts. Aberration‐corrected high‐angle annular dark‐field scanning transmission electron microscopy (HAADF‐STEM) detected scattered bright dots, suggesting that Cu species are anchored on the support surface in the form of isolated atoms or cations (Figure 1D,E), and the clear lattice fringe (*d* = 0.245) of the (100) crystal surface corresponded to Ti_3_C_2_. From the X‐ray diffraction (XRD) analysis depicted in Figure S4, only two wide peaks corresponding to Ti_3_C_2_ were observed, without any traces of Cu‐containing crystalline peaks (Figure S5), indicating the size of Cu is below the detection limit of XRD. X‐ray photoelectron spectroscopy (XPS) was adopted for investigating the electronic states of Cu single atoms, and the low‐resolution result displayed peaks for Ti, C, O, Cu and S elements (Figure 1F). The Cu2p XPS spectrum revealed prominent peaks at 932.4 and 952.4 eV, which were assigned to the +1‐chemical valence of Cu centers (Figure 1G). To further investigate the intricate electron structure and coordination surroundings of single Cu atoms within CuNTD, X‐ray absorption spectroscopy was performed on Cu K edge of this sample. As shown in Figure 1H, the spectra of X‐ray absorption at near‐edge structure (XANES) indicated that the energy edge distribution of single Cu atoms in CuNTD positioned close to Cu_2_O (between Cu foil and CuO). Linear mathematic simulation hinted that the copper species has an average chemical valence of +1, coincident with the findings of XPS, demonstrating predominant Cu(I) species. In Figure 1I, the Fourier‐transformed extended X‐ray absorption fine structure (EXAFS analysis of the coordination environment of Cu sites in CuNTD reveals a peak at 1.77 Å, indicating Cu‐S of the Cu‐NAC, with no detectable peak assigned to Cu─Cu bonding (2.27 Å), demonstrating Cu species exist as isolated atoms or cations in the CuNTD. Further digging into the EXAFS results in Figure 1J, only Cu─S binding was detected without Cu─Cu binding in the CuNTD, collaborating well with the HAADF‐STEM results. Figure 1K displays the EXAFS curve fitting of the CuNTD in k spaces, demonstrating high quality and convincing results. The fitting parameters of the above EXAFS result are listed in Table S1, suggesting one Cu atom is coordinated with two S atoms, forming S‐Cu(I)‐Cu sites. Wavelet transform (WT) study highlights the maximum intensity in CuNTD at 6.3 A^−1^, assignable to Cu─S bonds, while Cu foil exhibits the highest WT peak at 7.8 Å^−1^ attributed to metallic copper bonds (Figure 1L,M and Figure S6). Taken together, the XANES, EXAFS, and other characterization results strongly support our hypothesis that individual Cu atoms are bonded to NAC and are atomically dispersed on the CuNTD surface. These findings confirm the existence of Cu species as atomically dispersed entities with a stable chemical environment.

### 2.2 Evaluation of ROS Generation Performance

The TME exhibits elevated levels of H_2_O_2_ and GSH to maintain a distinct redox balance, while the nanozyme cascade catalyzed H_2_O_2_ and depletes GSH to produce ROS storms to kill tumor cells [25]. In addition, mild photothermal conditions can enhance the catalytic performance of enzyme‐like activities from the perspective of thermodynamics and increase tumor cell damage sensitivity [26]. Thus, we determined the photothermal and catalytic properties of CuNTD. The CuNTD exhibits good absorption behavior within near‐infrared region II (Figure S7), while the temperature of the CuNTD solution rises slowly as the concentration increases during irradiation (1064 nm laser, 1 W cm^−2^) (Figures S8 and S9A). Following this, the photothermal conversion efficiency (*η*) of CuNTD (54.21%) was determined utilizing the linear regression curve of the thermal cooling process shown in Figure S9C,D. Furthermore, negligible temperature fluctuation demonstrated the exceptional thermal stability of CuNTD through monitoring four cycles of irradiation (Figure S9B). These results indicate that CuNTD exhibits excellent photothermal properties, which could enhance catalytic activity.

**FIGURE 2:**
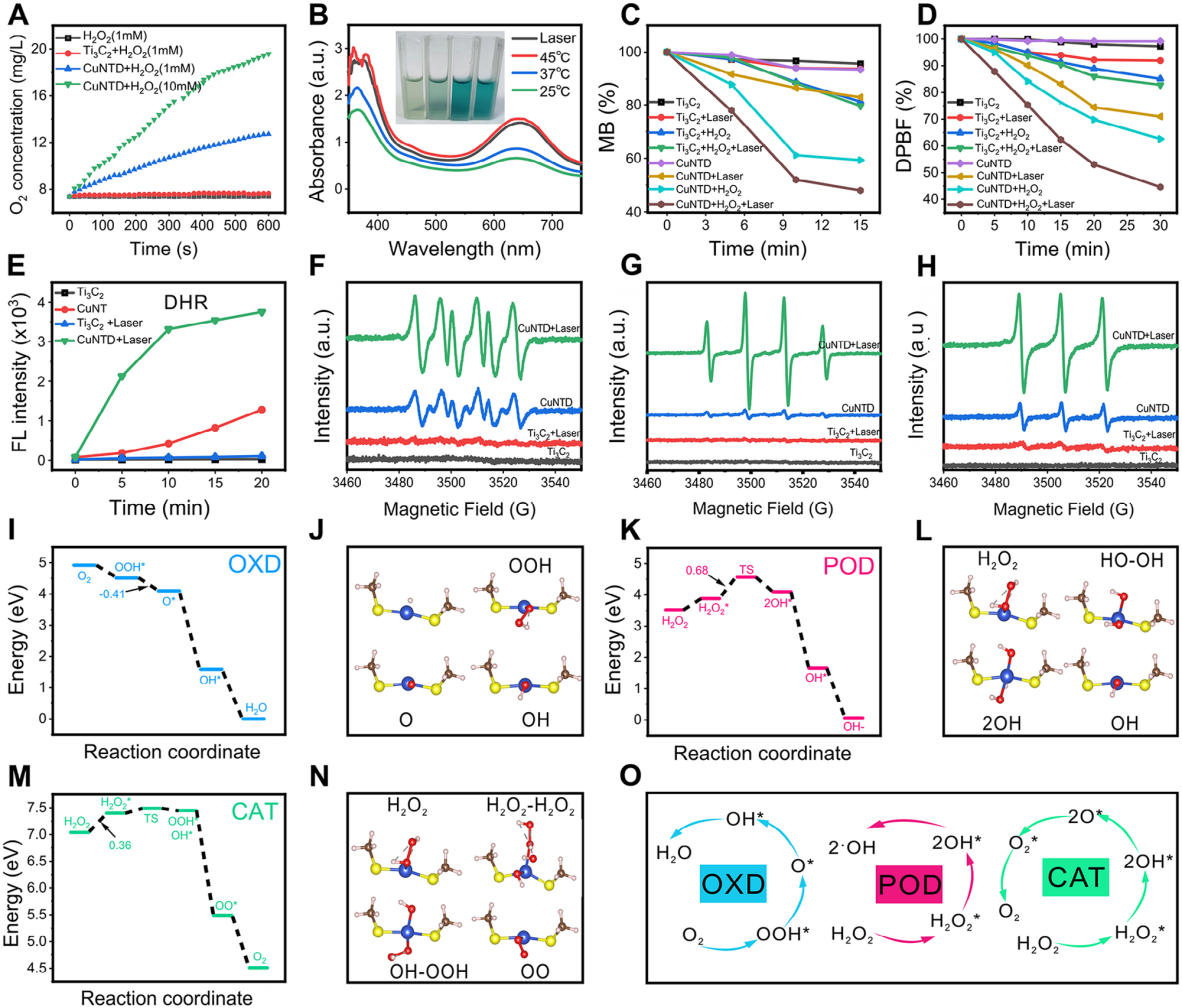
Enzyme catalytic performance test and DFT calculation. (A) O_2_ concentration curve of CuNTD (50 µg mL^−1^) without and with H_2_O_2_. (B) UV–visible absorption spectra of TMB (oxTMB) were measured at different treatments. (C) Proportion of MB degradation for Ti_3_C_2_ and CuNTD due to the generation of •OH containing H_2_O_2_. (D) Proportion of DPBF depletion due to ^1^O_2_ generation of Ti_3_C_2_ and CuNTD (laser on/off). (E) ROS detection using fluorescence intensity of DHR at different treatments. (F) ESR spectra of •OH trapped by DMPO, (G) •O_2_^−^ and (H) ^1^O_2_ trapped by TEMP were obtained with or without laser irradiation of Ti_3_C_2_ and CuNTD. (I–N) Proposed catalytic process resembling enzymes through DFT calculations. (O) Simplified description OXD‐, POD‐, and CAT‐like catalytic mechanism of CuNTD.

Then, the catalytic activity of CuNTD was evaluated. Initially, a portable dissolved oxygen meter was used for evaluating the CAT enzyme‐mimic performance of CuNTD. Figure 2A illustrates the capability of CuNTD to decompose H_2_O_2_ into O_2_, confirming its excellent CAT‐like activity and its potential to effectively alleviate tumor hypoxia, promoting ^1^O_2_ production through O_2_ [27]. Next, we detected the POD‐like catalytic properties of CuNTD using a colorimetric reaction involving 3,3′,5,5′‐tetramethylbenzidine (TMB) [3a]. The •OH generated by CuNTD catalyzing H_2_O_2_ oxidized colorless TMB into blue TMB (oxTMB), with typical absorbance peaks at 652 nm. Compared with unirradiated treatment, the oxTMB absorbance peak of the CuNTD group was increased after laser irradiation (Figure S10). To replicate the rise in temperature caused by the mild photothermal effect of CuNTD, we conducted reactions without 1064 nm laser irradiation in water bath at 25°C, 37°C, and 45°C, and compared them with reactions under 1064 nm laser irradiation (pH = 6.5). The oxTMB absorbance peak of CuNTD was increased with rising temperature and the laser‐irradiated curve closely resembles the 45°C treatment, suggesting that the photothermal effect boosts the efficiency of •OH generation by CuNTD (Figure 2B). Additionally, CuNTD's capacity to generate •OH was examined by methylene blue (MB) degradation (Figure 2C), showing a substantial reduction in MB content under laser irradiation, confirming the photothermal promotion of the catalytic process of producing •OH. The CuNTD's exceptional POD‐like activity was confirmed by the Michaelis–Menten constant (*K*_M_ = 9.31 mM) and maximum velocity (*V*max = 2.21 × 10^−7 ^M s^−1^) (Figure S11).

The feasibility of CuNTD producing singlet oxygen (^1^O_2_) was evaluated by the degradation of 1,3‐diphenylisobenzofuran (DPBF), depicted in Figure 2D. The photothermal effect was demonstrated by a sharp decrease in DPBF fluorescence with laser irradiation and H_2_O_2_ treatment, indicating enhanced production of ^1^O_2_ through the photocatalytic process [28]. Furthermore, the •O_2_^−^ production of CuNTD was also verified by •O_2_^−^ fluorescence probe dihydrorhodamine (DHR), and this process was enhanced by photothermal effect (Figure 2E). Subsequently, we employed electron spin resonance (ESR) spectroscopy to investigate the ROS produced by CuNTD. Figure 2F–H demonstrates a significant enhancement in •OH, •O_2_^−^ and ^1^O_2_ formation under laser irradiation compared to the non‐laser condition. In vitro, CuNTD exhibited photothermal‐enhanced multi‐enzyme capabilities, catalyzing H_2_O_2_ and O_2_ to produce highly toxic •OH, •O_2_^−^, and ^1^O_2_.

As mentioned above, GSH is an overexpressed endogenous antioxidant in TME and is also a critical endogenous copper chelator [29]. Therefore, GSH depletion can enhance oxidative stress and cuproptosis. Here, CuNTD can reduce GSH levels by utilizing 5,5'‐dithiobis (2‐nitrobenzoic acid) (DTNB) as a GSH hinter. The DTNB would react with ─SH groups to generate 2‐nitro‐5‐thiobenzoic acid anion (TNB^2−^), resulting in a bright, yellow‐colored reaction solution with a distinctive absorption peak at 412 nm. Figure S12 released that the intensity of the TNB^2−^ absorption peak gradually declined with CuNTD concentrations increase. Hence, these findings indicate that GSH‐depleting and photothermal‐enhanced cascade catalysis generates large amounts of ROS, thereby disrupting cell homeostasis and increasing the efficacy of ROS in killing tumor cells and inducing ICD.

Expanding on the findings from multi‐enzyme‐like reactions, we employed DFT calculations using the VASP code to uncover the potential catalytic pathways of OXD, POD, and CAT at the atomic scale of CuNTD (Figure 2I–O) [30]. Based on the experimental results, the enzymatic activities of CuNTD primarily depend on S‐Cu(I)‐S sites. Our attention was directed toward S‐Cu(I)‐S sites as the primary centers to investigate their microscopic electron configurations and the corresponding mechanisms of enzymatic catalysis. We created models for individual steps in the catalytic process and analyzed the energetic profiles to understand how changes in surface reactive sites affect the catalytic rate, considering the effects of surface adsorption, activation, dissociation, and desorption of CuNTD on all the intermediates. In POD‐like process, the initial three stages include H_2_O_2_ absorption and activation, culminating in the creation of the reactive *O and *OH. According to DFT calculations, the Cu center served as the potential active center, exhibiting a lower barrier (0.68 eV), which was overcome by the temperature elevation resulting from the photothermal effect of CuNTD. These findings imply that the catalytically active site in CuNTD is a copper atom with POD‐like behavior. Furthermore, a potential copper active site is also found on the sulfur‐copper‐sulfur surface for CAT‐like tasks. After oxidation and dehydration, the potential barrier of copper center decreased by 0.36 eV. Thus, confirming Cu atoms as the primary active centers in CuNTD, transforming the endogenous H_2_O_2_ into O_2_ molecules, further promoting the readily occurring OXD process for producing another toxic ROS‐•O_2_^−^. Thus, the bidirectional enzyme‐like transformation of H_2_O_2_ could simultaneously yield toxic ROS storms within the tumor cells and thus lead to high efficacy.

### 2.3 Cytotoxic Effect of CuNTD In Vitro

**FIGURE 3:**
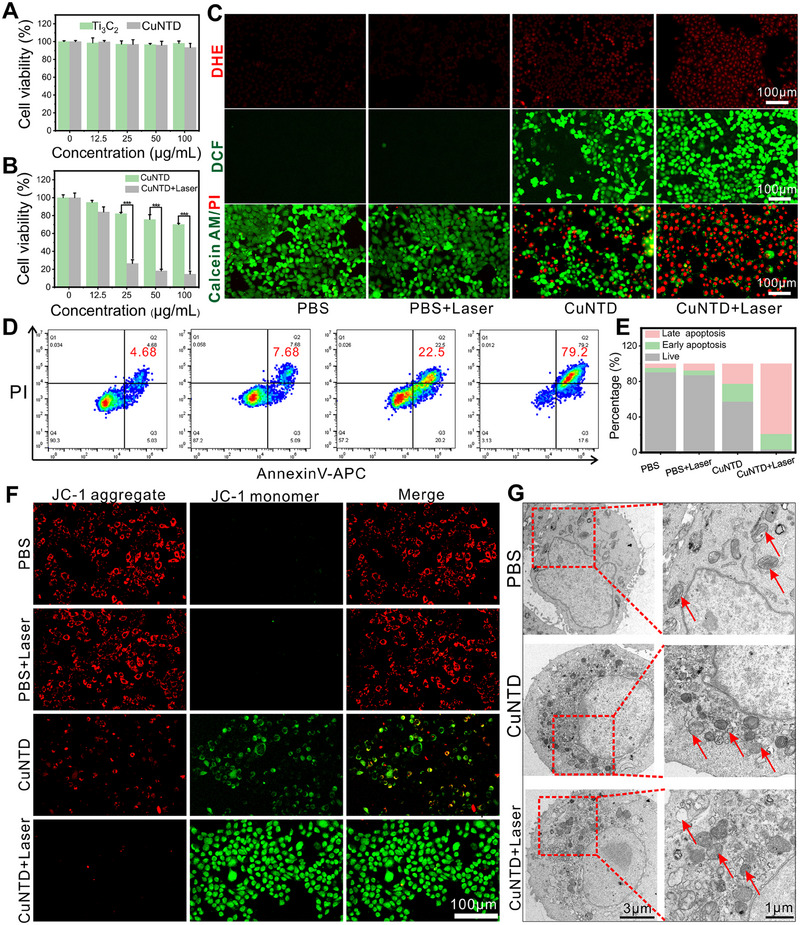
The antitumor activity and mechanism of CuNTD in cells. The viability of HUVEC cells (A) and 4T1 cells (B) when exposed to varying concentrations (0, 12.5, 25, 50, and 100 µg mL^−1^) of Ti_3_C_2_ and CuNTD (*n* = 3). (C) Fluorescence images of 4T1 cells labeled with DHE, DCFH‐DA and Calcein AM (green, live cells) and propidium iodide (red, dead cells). (D) Flow cytometry was used to analyze apoptosis by staining cells with annexin V‐FITC and PI following various treatments. (E) The corresponding quantification of cell apoptosis percentages. (F) Confocal images showing 4T1 cells stained with JC‐1. (G) Bio‐TEM images showing the effects of PBS and CuNTD treatment on 4T1 cells. (Irradiation conditions: 1064 nm, 1 W cm^−2^, 4 min). Statistical significance was determined using Student's *t* test: *p < 0.05, **p < 0.01, and ***p < 0.001.

Inspired by the noteworthy catalytic abilities of CuNTD, we tested the production of ROS and its anti‐cancer effects on the 4T1 cell line. The in vitro investigation focused on assessing the killing effect of CuNTD on cancer cells, with particular attention to its toxic and side effects on normal cells. Figure 3A shows that the survival rate of normal cells experienced a slight decrease in CuNTD increased, yet remained higher than 90%, indicating negligible toxicity. In contrast, the killing effect of CuNTD on 4T1 cells exhibited a gradual enhancement with increasing concentration, further intensified by laser irradiation (Figure 3B). The fluorescent probe dihydroethidium (DHE, a •O_2_^−^ probe) and 2', 7'‐dichlorodihydrofluorescein diacetic acid (DCFH‐DA, total ROS probe) staining tests were employed to evaluate intracellular ROS production (Figure 3C). In comparison to the control groups with almost no fluorescence, the CuNTD group showed weaker red fluorescence, while more obvious after irradiation treatment, indicating that CuNTD can produce •O_2_^−^ in cells through photothermal‐enhanced OXD‐like activity. Furthermore, intracellular ROS production induced by CuNTD was detected with the DCFH‐DA, and enhanced green fluorescence could be detected after being treated with laser irradiation. To visually demonstrate the killing effect on cells, calcein‐AM and propidium iodide (PI) staining were performed following different treatments. Figure 3C indicates a small fraction of dead cells were found in CuNTD groups, while almost all cells were destroyed in CuNTD with laser groups. Annexin V‐APC/PI staining (Figure 3D,E) revealed an apoptosis percentage of 42.7% for the CuNTD‐treated group, rising significantly to 96.8% with laser irradiation, corroborating the photothermal‐enhanced ROS killing effect.

Enhanced concentration of ROS leads to damage in the mitochondria, where multiple deadly signaling pathways come together [30]. For instance, cuproptosis is marked by mitochondrial lipid‐protein aggregation, while ferroptosis and apoptosis are associated with mitochondrial dysfunction, including the decline of mitochondrial membrane potential, fragmentation, as well as contraction. MMP is a crucial marker of mitochondrial damage, and JC‐1 serves as a fluorescence indicator to detect MMP. In Figure 3F, cells treated with CuNTD showed the highest green and the lowest red fluorescence, hinting severe damage to mitochondria compared to the control. Biological TEM was used to observe the morphological alterations of mitochondria following CuNTD treatment. As shown in Figure 3G, CuNTD caused severe damage to mitochondria, with a noticeable decline in mitochondrial size and higher membrane density, together with swelling and cavitation of the mitochondria. Therefore, the photothermal‐enhanced cascade catalytic effect of CuNTD generated ROS storms, efficiently killing 4T1 cells.

### 2.4 Multiple Death Mechanism Cascade Trigger ICD

**FIGURE 4:**
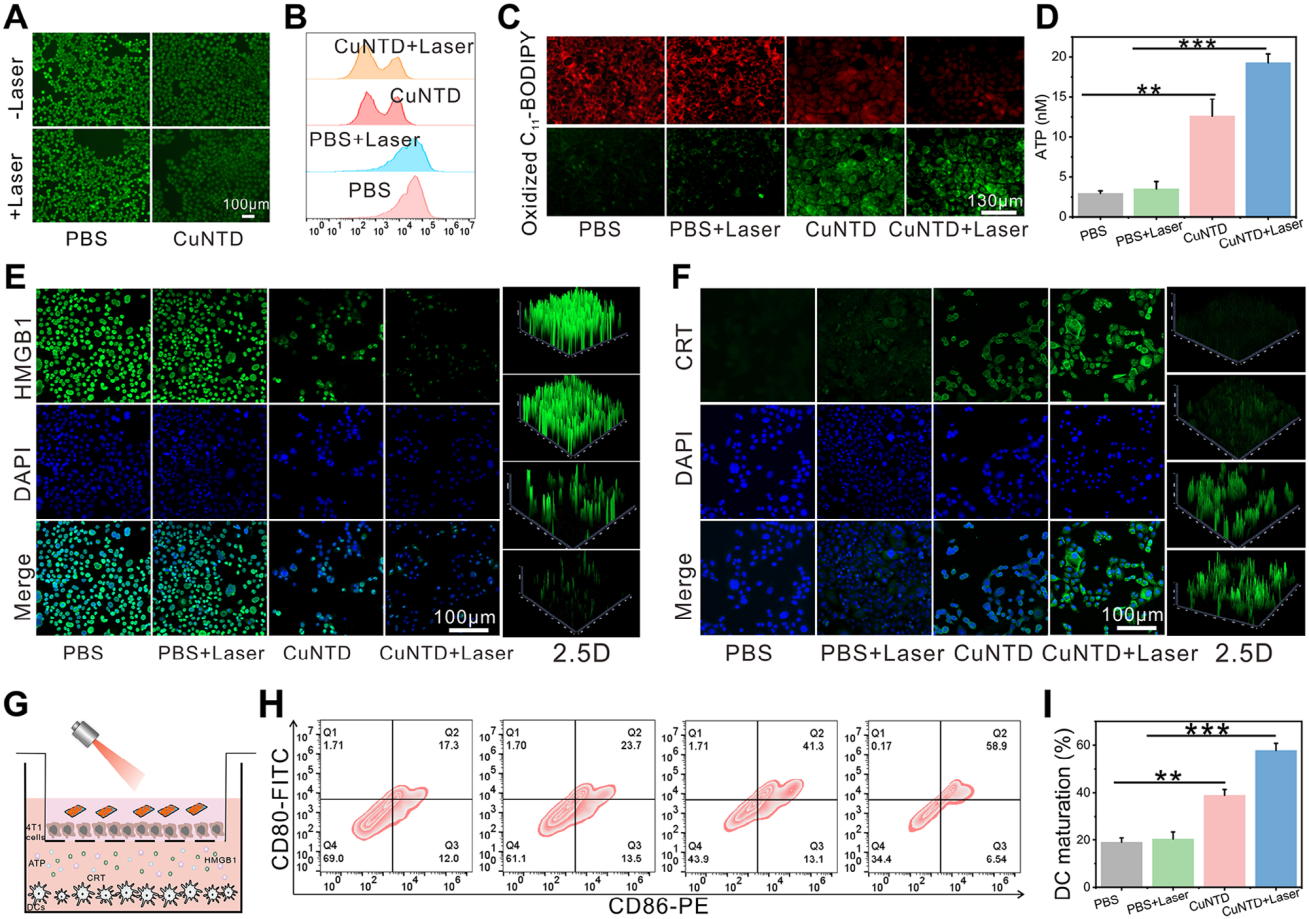
Studies on the mechanism of ROS storms, cuproptosis and ferroptosis cascade trigger ICD. (A) Fluorescence results of 4T1 cells labeled with thiol tracker violet following various treatments. (B) Flow cytometry data and their quantitative analysis were used to uncover intracellular GSH depletion. (C) Fluorescence results of C11_‐_BODIPY^581/591^ dye‐stained 4T1 cells. (D) ATP secretion in 4T1 cells (*n* = 3). CLSM images of CRT expression (E) and HMGB1 release (F) of 4T1 cells after different treatments. (G) Diagram showing transwell experiment. 4T1 cancer cells were added to the top chamber, while bone marrow‐derived dendritic cells were grown in the bottom chamber. (H) Flow cytometry analysis of BMDC maturation after coculturing with CuNTD with and without 1064 nm laser irradiation (1 W cm^−2^) in a transwell system. (I) Quantification of the DC maturation results in (K) (*n* = 3). Statistical significance was determined using the one‐way ANOVA: *p < 0.05, **p < 0.01, and ***p < 0.001.

As described above, GSH depletion directly disrupts intracellular antioxidant defenses to boost ROS accumulation [31]. In addition, GSH‐depletion not only enhances ICD in cancer cells but also can stimulate DC development and reduce immune suppression. A fluorescence probe for mercaptan was employed to assess the effect of CuNTD on intracellular GSH levels. Figure 4A demonstrates intense fluorescence in CLSM images of untreated 4T1 breast cancer cells, with a sharp reduction in fluorescence intensity following CuNTD addition. The flow cytometry also confirmed the above conclusions (Figure 4B). Additionally, the CuNTD was found to deplete intracellular GSH, as shown by an intracellular GSH assay kit. In comparison to the control groups, the levels of cellular GSH in CuNTD groups significantly decreased (Figure S13). These results suggested that CuNTD has an excellent ability to clear intracellular GSH.

Copper ions can be released when CuNTD is gradually oxidized by ROS and specifically degraded (Figures S14 and S15). Hence, we explored the newly discovered copper‐dependent cell death termed cuprotosis. Cu ions released act on lipoylated dihydrolipoamide S‐acetyltransferase (DLAT), inducing DLAT oligomerization. Elevated levels of insoluble DLAT result in proteotoxic stress, subsequently inducing cell death. Immunofluorescence staining were applied to substantiate our assumption. Figure S16 shows immunofluorescence imaging that supports the DLAT aggregation, with the highest protein aggregation fluorescence seen in the CuNTD group.

CuNTD oxidizes lipid peroxides (LPO) by producing •OH while inducing irreversible LPO through consumption of GSH, inactivating GPX4, effectively triggering ferroptosis characterized by LPO accumulation [29]. Therefore, LPO serves as a key biomarker of ferroptosis, and we utilized C11‐BODIPY as a proportional fluorescence index to detect LPO. Following oxidation, C11‐BODIPY's fluorescence transitioned from red to green, promoting the detection of LPO on the membrane. Notably, cells treated with CuNTD manifested intense green and weak red fluorescence, a process enhanced by photothermal effects (Figure 4C), indicating elevated lipid peroxidation levels. These results confirmed the CuNTD‐induced ferroptosis and this process was enhanced by photothermal effects due to promoting ROS production.

Then, we investigated the efficiency of CuNTD in inducing ICD by triggering the release of ATP, CRT, and HMGB1 through a photothermally enhanced ROS storm, cuproptosis and ferroptosis. Immunofluorescence staining revealed that 4T1 cells treated with CuNTD slightly increased CRT exposure and HMGB1 release (Figure 4E,F). However, in the CuNTD + Laser treatment group, CRT exposure and HMGB1 release were significantly increased. Additionally, ATP secretion by 4T1 cells substantially increased after CuNTD + Laser treatment (Figure 4D). These findings confirm that CuNTD + Laser treatment effectively induces ICD. Dendritic cells (DC), which are essential antigen‐presenting cells (APCs) and serve as the starting point for both innate and adaptive immunity, play a vital role. Because these DAMPs can offer adjuvant stimulation for DCs, we assessed the maturation of DC by co‐culturing bone marrow‐derived dendritic cells (BMDCs) with 4T1 cells in the Transwell system for 24 h, as illustrated in Figure 4G. Flow cytometry was employed to confirm the presence of CD80 and CD86 markers, indicating the maturation of DCs (Figure 4H,I). The CuNTD‐treated groups effectively enhanced the maturation of DCs to approximately 41.3%, reaching a level about ≈3.4‐fold of the control groups. Furthermore, CuNTD with laser group has further enhanced the maturation of DCs.

### 2.5 Proteomic Analysis of CuNTD in 4T1 Cells

**FIGURE 5:**
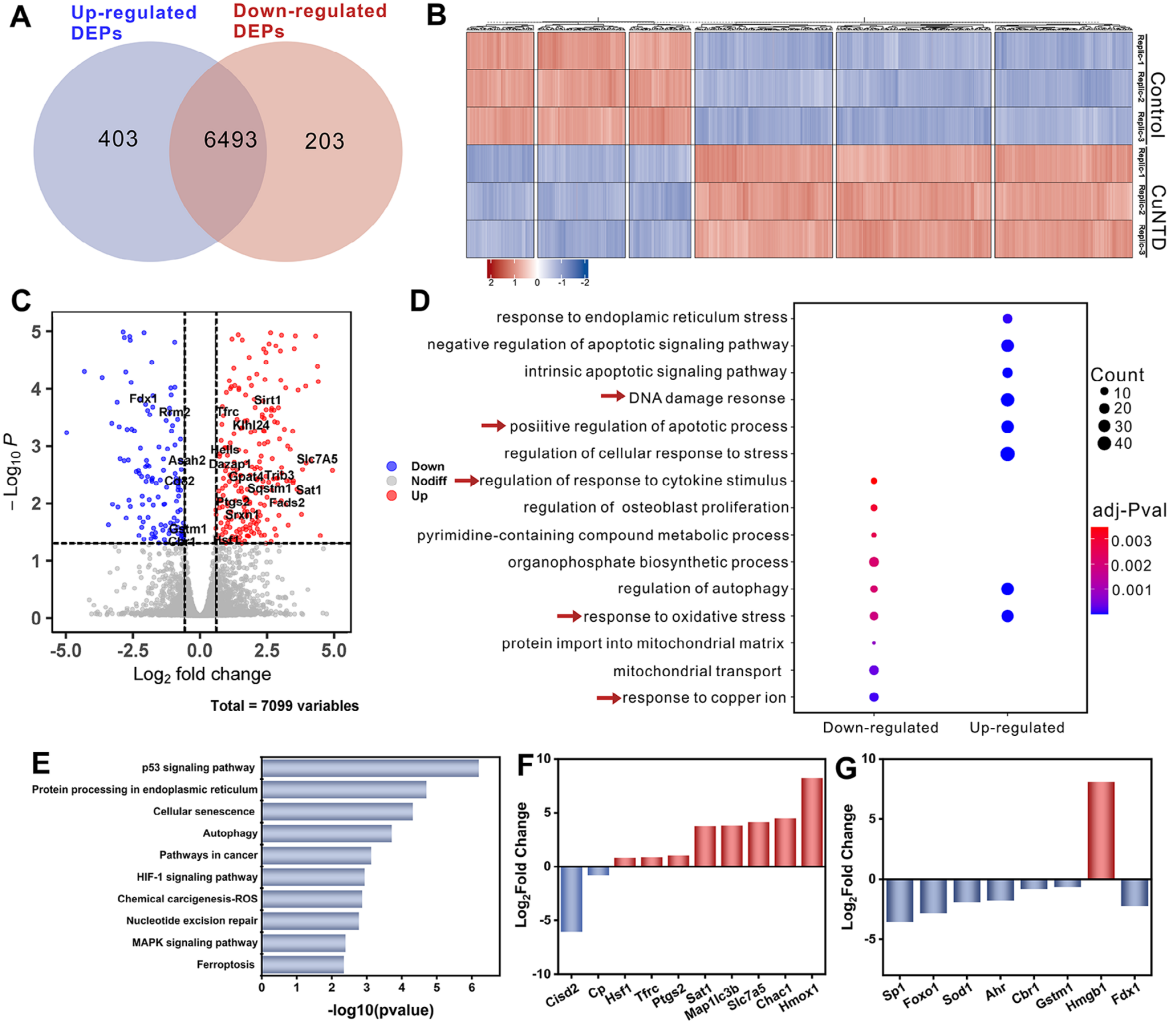
Quantitative proteomics analysis of 4T1 cells under different treatment conditions. (A) Venn diagram of identified DEGs in pairwise comparison. (B) Heatmap showing the DEPs between CuNTD in comparison to the PBS group. (C) Volcano plot showing the upregulated and downregulated proteins CuNTD compared to the PBS group. (D) Dot plot displaying the biological pathways differentially regulated by CuNTD in comparison to the PBS group using GO enrichment analysis. (E) Bar plot indicating the pathways differentially regulated by CuNTD compared to the PBS group using KEGG pathway enrichment analysis. The proteins that were up‐regulated and down‐regulated were examined individually. (F,G) Bar plot displaying the alterations in expression levels of crucial proteins related to ferroptosis, cuproptosis, ROS response and ICD induction.

To elucidate the molecular mechanisms underlying the action of CuNTD, we conducted a quantitative proteomic study to investigate the changes in protein expression in 4T1 tumor cells following CuNTD treatment [32]. Two sets of 4T1 cell samples were collected for each treatment. Liquid chromatography‐tandem mass spectroscopy (LC‐MS/MS) identified a total of 7099 proteins. This analysis revealed 606 differentially expressed proteins (DEPs) between CuNTD and the PBS group, including 403 upregulated and 203 downregulated proteins (FC > 2, *p* < 0.05) (Figure 5A–C). As anticipated, the GO biological process enrichment analysis and KEGG pathway analysis revealed abnormalities in cell cycle progression and mitotic nuclear division following CuNTD treatment, aligning with the observed cytotoxic effects. Furthermore, CuNTD‐treated cells showed enrichment of pathways related to ROS response, apoptosis, cuproptosis, and ferroptosis (involving lipid and GSH metabolic processes) in comparison with the PBS group (Figure 5C,D and Figure S17).

Within the differentially expressed proteins, there was an increase in the concentration of p53, as shown in Figure 5E. p53, known as the protector of the genetic material, is essential for regulating cell cycle inhibition, aging, and programmed cell death. Demonstrating its ability to block cysteine absorption, its activation makes cells more susceptible to ferroptosis. Additionally, the enzyme heme oxygenase‐1 (HMOX1), which facilitates the oxygen‐dependent breakdown of heme into biliverdin, carbon monoxide and Fe^2+^, was upregulated upon CuNTD treatment (Figure 5F). Although HMOX1 can provide cell protection from different stress‐related situations, elevated amounts have been linked to the triggering of ferroptosis. The enzyme prostaglandin G/H synthase 2 (Ptgs2, or Cox2) showed a notable increase in activity, suggesting the start of ferroptosis due to its combined cyclooxygenase and peroxidase functions. Moreover, the transferrin receptor protein 1 (Tfrc, or TfR1), which plays a crucial role in cellular iron absorption, exhibited elevated levels following CuNTD treatment, indicating the activation of the ferroptosis pathway.

After exposure to CuNTD, the expression of various GSH S‐transferase proteins related to GSH metabolism, such as Gstm1, was notably reduced in the cellular response to ROS. GST proteins, part of the phase II detoxification enzymes, contribute to chemoresistance development, and their downregulation could enhance ROS‐mediated oxidative damage. Additionally, as shown in Figure 5G, Sod1, an intracellular enzyme that helps convert superoxide anion into oxygen and H_2_O_2_, was also decreased, worsening ROS signaling and harm. HMGB1 was implicated in triggered ICD, and Fe–S cluster proteins ferredoxin (FDX1) served as a key regulator of cuproptosis. These findings highlight that CuNTD treatment primarily induced cancer cell death by activating apoptosis, ferroptosis, cuproptosis, and ROS storms, which subsequently initiated cascade‐triggered ICD.

### 2.6 In Vivo Antitumor Therapy of CuNTD

**FIGURE 6:**
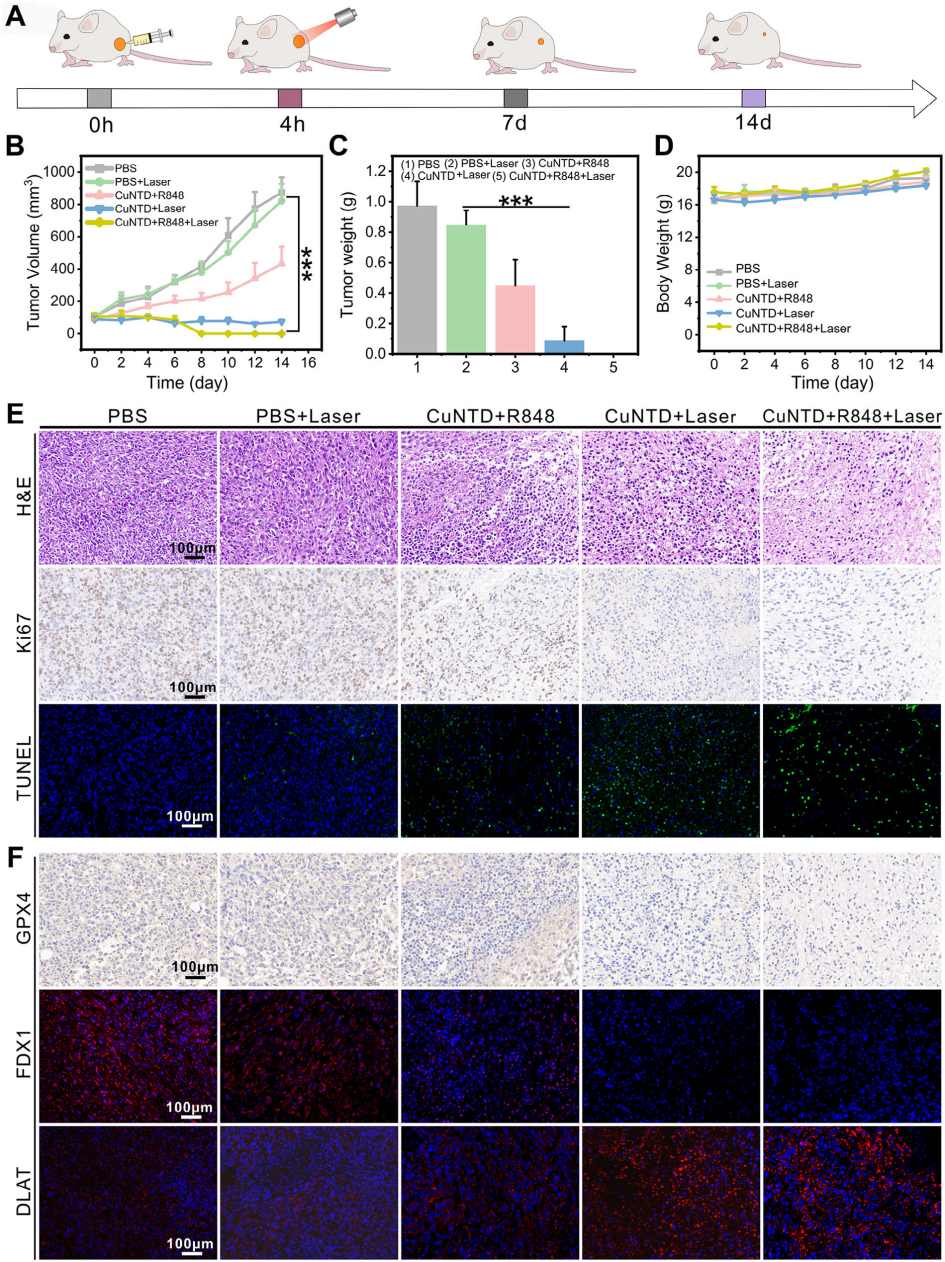
The antitumor therapy of CuNTD in vivo. (A) A diagram was used to show the process of establishing the 4T1 tumor model and the treatment schedule after i.t. administration. (B) Tumor volume curves, (C) tumor weights and (D) body weights plots in different groups (*n* = 5). (E,F) Images of tumor slices stained with H&E, TUNEL, Ki67, GPX4, FDX1, and DLAT 24 h after treatments. Statistical significance was determined using the one‐way ANOVA: *p < 0.05, **p < 0.01, and ***p < 0.001.

In terms of the aforementioned findings, we established a 4T1 tumor‐bearing mouse model to evaluate the in vivo antitumor efficacy of CuNTD (Figure 6A). Given that CuNTD cascade triggered the ICD of tumor cells to promote DC maturation to stimulate a stronger immune response, we combined CuNTD with co‐stimulatory immune adjuvant R848 in mouse tumor therapy. Initially, when the tumor inoculation on the back of mice reached around 100 mm^3^, the mice were allocated randomly to PBS, PBS + Laser, CuNTD + R848, CuNTD + Laser, and CuNTD + R848 + Laser groups. The nanomedicine was injected in situ into the tumor site. Subsequently, to ensure optimal absorption of the nanocomposite by tumor cells, the tumor region was exposed to laser irradiation (1064 nm, 0.5 W cm^−2^) for 4 min to ensure the temperature was around 45°C, 4 h after it was administered. Infrared thermal imaging camera was employed to record the temperature distributions in mice in real‐time and spatially (Figure S18). These findings showed the temperatures at tumor sites of CuNTD group and CuNTD + R848 group were significantly higher compared to PBS group, suggesting that CuNTD still has good photothermal effects to enhance multi‐mechanisms therapy in vivo.

Figure 6B and Figure S19 indicated that the tumor volume of mice in PBS + Laser group exhibited a similar trend to that in the PBS group, displaying rapid growth. In contrast, the CuNTD + R848, CuNTD + Laser, and CuNTD + R848 + Laser groups demonstrated moderate inhibition, with more pronounced effects observed after near‐infrared irradiation. Notably, the CuNTD + R848 + Laser group completely eradicated the tumor, showing a robust therapeutic effect of photothermally promoted multi‐mechanisms therapy. After treatment, the superior antitumor effect of CuNTD + R848 was confirmed through the assessment of excised tumor weight (Figure 6C) and the acquisition of corresponding tumor images (Figure S20).

To delve into the specific therapeutic mechanisms of this material, tumor tissue was collected 24 h post laser treatment and subjected to hematoxylin and eosin staining (H&E), Ki67 antibody staining, as well as terminal deoxyriboside transferase dUTP incision end labeling (TUNEL) (Figure 6E). H&E staining revealed severe damage to tumor cells in CuNTD + R848 group in comparison with the PBS group following laser irradiation. Ki67 antibody staining demonstrated that CuNTD treatment inhibited the proliferation activity of tumor cells, whereas the PBS group exhibited almost no adverse effect on cell proliferation. TUNEL staining identified a substantial number of TUNEL‐positive cells, indicative of destructive necrosis and apoptosis in tumor cells. Conversely, the other treatment groups showed minimal or no significant tumor damage.

Furthermore, immunofluorescence was used to detect the expression of GPX4, FDX1 and DLAT in tumors (Figure 6F). Consistent with in vitro findings, the greatest pronounced DLAT aggregation and loss of FDX1 were seen in tumors exposed to CuNTD + R848 + Laser, providing evidence of cuprotosis occurring in vivo. Furthermore, tumors treated with CuNTD + R848 + Laser displayed the most pronounced GPX4 downregulation, affirming the effectiveness of CuNTD in inhibiting tumor growth through cuproptosis and ferroptosis.

Notably, we assessed the possible systemic toxicity of CuNTD by examining hemocompatibility, blood biochemical analysis, weight changes and histological analysis of key organs in mice. The hemolysis assay suggested the value of CuNTD was below the threshold of 5%, indicating its good blood compatibility (Figure S21). The blood biochemical analysis also illustrated that CuNTD had good biosafety (Figure S22). The mice maintained a consistent body weight following various treatments, as shown in Figure 6D, and no major organ damages were detected in the heart, liver, spleen, or kidney, as Figure S23 illustrates. These analyses illustrated that either CuNTD or CuNTD + R848 had good biosafety. Together, the data consolidate an admirable synergistic anti‐tumor performance of CuNTD in vivo.

### 2.7 In Vivo Antitumor Immunotherapy of CuNTD

**FIGURE 7:**
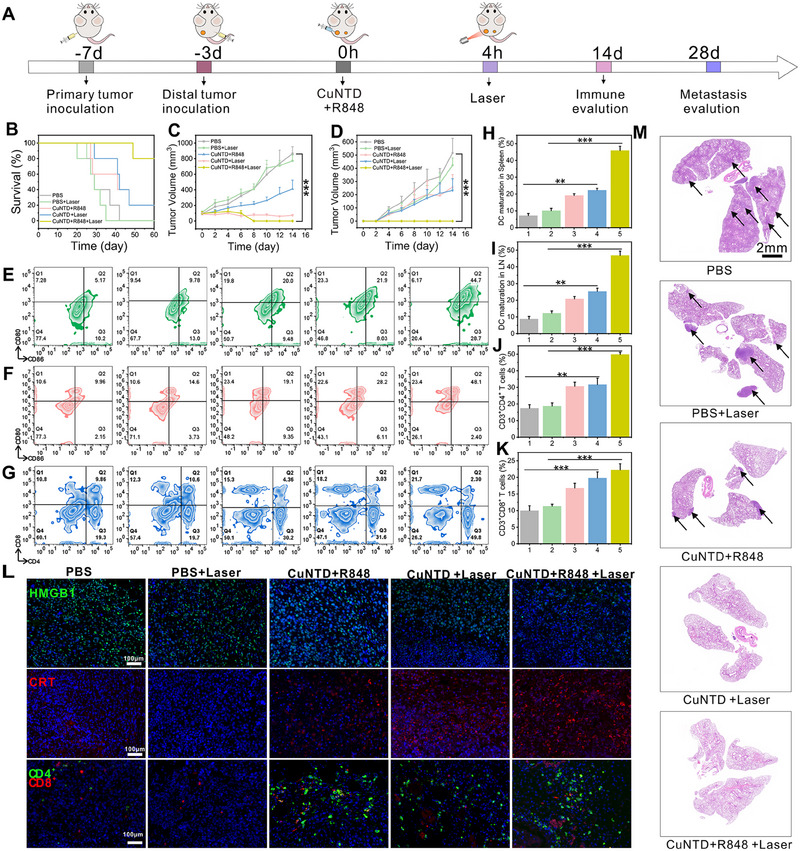
Studies on the antitumor immunotherapy of CuNTD in vivo. (A) The schedule of in vivo combined therapy to restrain bilateral tumor recurrence and metastasis. (B) Long‐term monitoring of Balb/c mice post‐treatment shows their survival rate. (C,D) Sizes of the primary and distant tumors (*n* = 5). Flow cytometry was used to analyze CD11c^+^CD80^+^CD86^+^ DCs in lymph nodes (E) and in spleen (F), CD3^+^CD4^+^ T cells and CD3^+^CD8^+^ T cells in spleens (G) following treatments (1:PBS, 2:PBS + Laser, 3:CuNTD + R848, 4:CuNTD + Laser, 5:CuNTD + R848 + Laser). The quantification of CD80^+^CD86^+^ DCs in lymph nodes (H) and in the spleen (I), CD3^+^CD4^+^ T cells (J) and CD3^+^CD8^+^ T cells (H) in spleens following different therapies. (L) Immunofluorescence staining of HMGB1 (green), CRT (red), CD8^+^ T cells (green) and CD4^+^ T cells (red) from primary tumor tissues of mice. DAPI was used to stain cell nuclei with blue fluorescence. (M) H&E staining result of representative lung regions for antimetastatic research, and the enlarged analysis is in the Supporting Information S22. Statistical significance was determined via the one‐way ANOVA: *p < 0.05, **p < 0.01, and ***p < 0.001.

Following the highly effective of tumors in situ with minimal side effects of normal tissue, we evaluated the in vivo immunotherapeutic performance of CuNTD to assess its efficacy in inhibiting tumor metastasis and distal tumors. The activation of immune response by CuNTD combined with R848 cascade‐triggered immunotherapy was further investigated by examining mouse survival, ICD induction, DCs maturation, T cell initiation, and lung metastasis. Using a bilateral 4T1 tumor model, mice were injected with the distal tumor four days following the formation of the primary tumor and subsequently exposed to various samples (Figure 7A). Figure 7C,D demonstrates that the CuNTD + R848 group successfully eliminated the main tumor and inhibited the distant tumor, showing encouraging immune capabilities.

The lymph nodes and spleen are known to serve as crucial components in immune systems, housing numerous lymphocytes and serving as the primary hubs for the body's defense mechanisms. Therefore, we collected the lymph nodes and spleen of mice and evaluated the DC maturation and the population of T cells using flow cytometry. Figure 7E,H demonstrates that the DC maturation proportions of lymph nodes for the CuNTD + R848 + Laser group (44.7%) were 2‐, 2.2‐, 4.6‐ and 8.6‐fold higher than the CuNTD + Laser (21.9%), CuNTD + R848 group (20%), PBS + Laser group (9.78%) and PBS group (5.17%), respectively. Then, the maturation of DCs was also examined in the spleen (Figure 7F, I). The DC maturation levels for the CuNTD + R848 + Laser group (48.1%) were 1.7‐, 2.5‐, 3.3‐ and 4.8‐fold higher than CuNTD + Laser group (28.2%), CuNTD + R848 group (19.1%), PBS + Laser group (14.6%) and PBS group (9.96%), respectively (Figure 7F). This observation suggests that CuNTD + R848 can effectively activate DC cell maturation, which will subsequently stimulate a strong activation of T cell immunity (Figure 7G,J,K). Cytotoxic T lymphocytes (CD8^+^ T cells) and helper T cells (CD4^+^ T cells) are crucial in directly combating cancer cells and are necessary for controlling adaptive immunity[22b]. Thus, the proportion of T cells in the spleen was subsequently measured. The percentage of activated CD3^+^ CD4 ^+^ in the CuNTD + R848 + Laser group (49.8%) was 1.6‐, 1.6‐, 2.5‐ and 2.6‐fold higher than CuNTD + Laser group (31.6%), CuNTD + R848 group (30.2%), PBS + Laser group (19.7%) and PBS group (19.3%) respectively. In addition, the percentage of cytotoxic T lymphocytes (CD3^+^CD8^+^, 21.7%) manifests 1.2‐, 1.4‐, 1.8‐ and 2‐fold increase in population compared to CuNTD + Laser group (18.2%), CuNTD + R848 group (15.3%), PBS + Laser group (12.3%) and PBS group (10.8%), respectively. Overall, the findings suggest that multi‐mechanism therapy can effectively trigger ICD to activate a strong immune response, which is a crucial step in augmenting anti‐tumor immunity.

Immunofluorescence staining was used to examine the presence of key biomarkers associated with ICD (HMGB1 and CRT) in tumor samples. These results revealed that HMGB1 expression was prominently decreased (Figure 7L) in the nucleus of CuNTD + R848 + laser group, and the exposure of CRT was increased. Additionally, the CuNTD + R848 + Laser group exhibited the largest population of CD4^+^ helper T cells and CD8^+^ cytotoxic T cells in tumor regions, as confirmed through immunofluorescence staining (Figure 7L). Simultaneously, no recurrence of the distal tumor was observed during the treatment period, leading to a substantially improved survival rate (Figure 7B). This finding consolidated the sustained long‐term immune effect. In addition, the lung metastasis inhibition effect of typical metastatic cancer therapy was validated by H&E staining (Figure 7M and Figure S24). Notably, after 28 days of synergistic treatment, there were nearly no metastatic nodules compared to the control group.

Collectively, the findings manifested that under laser irradiation CuNTD combined with R848 could effectively induce an immune response against cancer via promoting DC maturation and activating T cells, ultimately offering a strong defense against tumor recurrence as well as metastasis.

## 3 CONCLUSION

In conclusion, we successfully anchored single S‐Cu(I)‐S sites uniformly onto the surface of 2D Ti_3_C_2_ a straightforward coordination self‐assembly strategy. This process enabled the fabrication of atomically dispersed multifunctional copper(I) nanomodulator (CuNTD). CuNTD was employed to induce strong ICD through multiple regulatory pathways of cascade photothermal‐amplified ROS storms, cuproptosis, and ferroptosis, thus effectively promoting DC maturation while mitigating the side effects and resistance associated with monotherapies. EXAFS and HAADF‐STEM experiments verified the dispersion of copper in the form of single atoms on the Ti_3_C_2_ surface, with a high weight loading of 1.5 wt% determined by ICP. In vitro enzyme‐activity tests and DFT calculations demonstrated that CuNTD exhibited cascade CAT‐like, OXD‐like, and POD‐like activities, along with mild photothermal‐enhanced enzymatic activities. This led to the efficient generation of •O_2_^−^, ^1^O_2_, and •OH. Subsequent in vitro experiments confirmed that CuNTD catalyzed the overexpression of H_2_O_2_ and depletion of GSH in TME, generating ROS storms that lead to mitochondrial dysfunction, lipid peroxidation‐mediated ferroptosis, and release of Cu causing DLAT aggregation‐mediated cuproptosis. Furthermore, strong ICD induced release of damage‐associated molecular patterns (ATP, HMGB1, and CRT) from tumor cells, effectively activating the maturation of DC cells and CD8^+^ T cell infiltration. In vivo studies demonstrated that CuNTD exhibited high efficacy for treating primary tumors and successfully inhibiting distant tumors and metastatic lesions through cascade‐triggered immunotherapy. Moreover, the combination with the immune adjuvant R848 enhanced antigen presentation formed immune memory, and significantly improved mice survival rate. Therefore, we introduce CuNTD as a novel, biocompatible, and efficient ICD inducer for triple‐negative breast cancer (TNBC) immunotherapy, leveraging multiple cell death mechanisms. This work highlights the potential of structurally well‐defined and highly functional copper‐based nanomodulators, contributing to the advancement of precise nanomaterials in TNBC immunotherapy.

## 4 Methods

### 4.1 Synthesis of CuNTD

First, 6.2 mg copper acetate and 10 mg *N*‐acetylcysteine were combined to form a complex with a molar ratio of 1:2 (Cu/NAC) solution under sonication for 10 min. Next, the Cu‐NAC was added into the Ti_3_C_2_ nanosheets (20 mg) tris buffer under magnetic stirring for 1 h to obtain CuNT based on the coordination ligand‐driven self‐assembly. CuNTD aqueous solution was washed three times under sonication to remove the unloaded Cu‐NAC complex. Then, DSPE‐PEG2000 solution was added to the above solution, stirred for 2 h, and the as‐obtained CuNTD was collected and washed by centrifugation.

### 4.2 In Vitro DCs Maturation

To investigate in vitro maturation of DC, we collected bone marrow‐derived DC from the femur of 6‐week Balb/c female mice. Usually, bone marrow is harvested, treated with red blood lysate buffer to eliminate red blood cells, and then cultured at 37°C in a medium containing granulocyte‐macrophage colony‐stimulating factor (GM‐CSF, 20 ng mL^−1^) and IL‐4 (10 ng mL^−1^). On day 7, immature dendritic cells were collected for further experiments. 4T1 cells were seeded into the upper compartments of the transwell while immature DCs were seeded into the lower compartments of the same transwell. Following a 24‐h incubation period, 4T1 cells were treated with CuNTD, followed by irradiation 4 h later. Subsequently, staining was conducted using anti‐CD80 and anti‐CD86 antibodies, and flow cytometry was utilized to determine the proportion of mature DCs in the lower compartments.

### 4.3 Animal Model

All animal experiments were performed according to the guidelines approved by the Institutional Animal Care and Use Committee of the First Affiliated Hospital of Shenzhen University (Shenzhen) (Approval number: 20220098). Female Balb/c mice (16–18 g, 6 weeks old) were randomly divided into five groups (*n* = 6 per group). 1 × 10^6^ 4T1 cells were subcutaneously injected into the right back of Balb/c mice. When the tumor became distinct and the tumor volume reached about 100 mm^3^, the mice were randomly assigned into either control or test groups. To establish a bilateral tumor model, 4T1 cells were injected subcutaneously into the left (primary tumor) and a distal tumor was constructed on the right 4 days later.

### 4.4 In Vivo Cancer Therapy

The 4T1 tumor‐bearing mice were randomly divided into five groups *(n* = 8). Subsequently, the various categories received specific interventions in the following manner: (1) PBS, (2) PBS + Laser, (3) CuNTD + R848, (4) CuNTD + Laser, and (5) CuNTD + R848 + Laser. The treatment groups were administrated by intratumor injection of PBS, CuNTD and CuNTD+R848, respectively. The injection dose was controlled at 15 mg/kg. The tumor site was irradiated with a laser (1064 nm, 0.5 W cm^−2^) for 4 min after 4 h of injection. The real‐time temperature changes at the tumor region were recorded by an infrared thermography. Drug administration and irradiation treatment were performed only once. Body weight and tumor volume (tumor volume = length × width^2^/2) were monitored in mice with tumors every other day using a vernier caliper and electronic scale. Following a 14‐day treatment period, the mice were euthanized, and their tumor samples were collected and measured. Photographs were taken of the tumors in each group for comparison, and the major organs' tissues were stained with H&E. To investigate the process of tumor therapy, three mice from each group were randomly euthanized 24 h after laser exposure. H&E staining, TUNEL staining, Ki67 antibody staining, ferroptosis protein GPX4, cuproptosis protein (DLAT, FDX1) immunofluorescence staining was performed on tumor tissues.

### 4.5 Statistical Analysis

The data are presented as the mean ± standard deviation (SD), and the statistical significance between two groups of data in this work was analyzed based on a two‐tailed Student's *t*‐test or one‐way ANOVA for multiple comparisons. (* *p* < 0.05; ** *p* < 0.01; *** *p* < 0.001).

## Author Contributions

**Yuanyuan Zhang**: writing – original draft, validation, investigation, methodology, project administration, visualization, formal analysis, data curation, conceptualization. **Shengnan Ya**: writing – review and editing, methodology, supervision, resources, conceptualization. **Jingnan Huang**: visualization, software, methodology, formal analysis. **Yangyang Ju**: visualization, software, methodology. **Xueyang Fang**: resources, methodology, formal analysis. **Xinteng Ouyang**: investigation, software. **Qingdong Zeng**: methodology, formal analysis. **Xinyao Zhou**: writing – review and editing. **Xiyun Yan**: resources, project administration. **Guohui Nie**: visualization, resources, project administration, funding acquisition. **Kelong Fan**: writing – review and editing, supervision, resources, funding acquisition, conceptualization. **Bin Zhang**: writing – review and editing, supervision, resources, project administration, funding acquisition, formal analysis.

## Conflicts of Interest

The authors declare no conflicts of interest. Kelong Fan is a member of the *Exploration* editorial board, and he was not involved in the handling or peer review process of this manuscript.

## Supporting information



Supporting Information

## Data Availability

Data will be made available on request.
