# Supplementary material for: Spatial Isolation of Single Copper(I) Sites for Cascade Enzyme‐Like Catalysis and Simultaneous Ferroptosis/Cuproptosis Boosted Immunotherapy
Source: Exploration (Beijing). 2025 Mar 6;5(3):20240275. doi: 10.1002/EXP.20240275 (PMC12199402; doi:10.1002/EXP.20240275)
Supplement: Supplementary file 1 — Supporting Information [file EXP2-5-20240275-s001.docx]

**Supplementary Information**

**Spatial Isolation of Single Copper(I)** **Sites for** **Cascade Enzyme-like Catalysis and Simultaneous** **Ferroptosis/Cuproptosis Boosted** **Immunotherapy**

*Yuanyuan Zhang^a^, Shengnan Ya^c^, Jingnan Huang^d^, Yangyang Ju^a^, Xueyang Fang**^a^, Xinteng Ouyang^a^, Qingdong Zeng^a^, Xinyao Zhou^f^, Xiyun Yan^b,e^, Guohui Nie^a,^*, Kelong Fan^b,e,^*, Bin Zhang^a,^**

^a^Shenzhen Key Laboratory of Nanozymes and Translational Cancer Research, Department of Otolaryngology, Shenzhen Institute of Translational Medicine, The First Affiliated Hospital of Shenzhen University, Shenzhen Second People's Hospital, Shenzhen University Medical School, Shenzhen, 518035, China.

^b^CAS Engineering Laboratory for Nanozyme, Key Laboratory of Biomacromolecules (CAS), CAS Center for Excellence in Biomacromolecules, Institute of Biophysics, Chinese Academy of Sciences, Beijing, 100101, China.

^c^School of Medical Imageology, Wannan Medical College, Wuhu, 241002, China.

^d^Department of Gastroenterology, The First Affiliated Hospital (Shenzhen People's Hospital), Southern University of Science and Technology, Shenzhen, 518055, China.

^e^Nanozyme Laboratory in Zhongyuan, Zhengzhou, Henan, 451163, China.

fFischell Department of Bioengineering, University of Maryland, College Park, Maryland, 20740, USA.

*Corresponding authors

1. mail: [binzhang@email.szu.edu.cn](mailto:binzhang@email.szu.edu.cn),

[fankelong@ibp.ac.cn](mailto:fankelong@ibp.ac.cn),

[nieguohui@email.szu.edu.cn](mailto:nieguohui@email.szu.edu.cn,).

**Supplementary Methods**

**Materials**

Copper acetate was purchased from Aladdin (China), N-acetylcysteine was purchased from Beyotime (China), DSPE-PEG2000 was purchased from Xi’an ruixi Biological Technology Co., Ltd. (China). All chemicals were used directly without further purification. All chemicals were used directly without further purification.Thermo Fisher Scientific Corporation supplied all the media used for cell culture.

**Characterization of CuNTD**

The CuNTD was analyzed using TEM and element mapping on a FEY-TalOS F200X microscope with an acceleration voltage of 200 kV (FEI, USA). GeminiSEM 300 from Zeiss in Germany was used to perform scanning electron microscopy (SEM) at 5 keV. The JEM ARM 200F instrument from JEOL in Japan was utilized to conduct HAADF-STEM at 200 kV acceleration voltage. XRD was carried out using the D8 Advance instrument produced by Bruker, Germany, with a 2θ range of 3-30 degrees. XPS data was collected using an Escalab 250Xi instrument from Thermo Scientific in the United States. The generation of ROS was measured by an electron spin resonance (ESR) spectrometer (Bruker EMXplus). Inductively coupled plasma emission spectrometry (ICP-OES Avio 200) was used to analyze the levels of Cu and Ti in the samples. EXAFS was performed under the 21A beam of the Taiwan photon Source. The four-reflection channel cut Si (111) monochromator is used for single-beam X-ray absorption spectroscopy. At the final stop, there are three ionization chambers and Lytle/SDD type detectors for both transmission and fluorescence modes of XAFS. The monochromatic photon flux on the sample ranges from 6 to 27 keV, about 1 × 10^11^ to 3 × 10^9^ photons /s.

**CAT-like Activity of** **CuNTD**

CAT activity assays of CuNTD were detected by measuring the oxygen concentration produced. Different amounts of CuNTD and H_2_O_2_ were introduced into 10 mL of PBS with a pH of 7.4. The levels of oxygen were monitored every 15 seconds for a total of 10 minutes using a dissolved oxygen meter.

***In Vitro* Photothermal Performance of** **CuNTD**

The photothermal efficiency was assessed by exposing the CuNTD aqueous solution to a 1064 nm laser (1 W cm^-2^) for 300s at room temperature. The temperature variation and thermal picture were captured and documented using an IR thermal camera at various intervals. Similarly, PBS was exposed to radiation in identical conditions. Different levels of CuNTD water-based mixture (ranging from 0 to 100 μg mL^-1^) were exposed to a 1064 nm laser at a power density of 1 W cm^-2^ for a duration of 5 minutes. The CuNTD solution in water was exposed to radiation at a power density of 1 W cm^-2^ in order to determine the efficiency of photothermal conversion. The temperature was measured every 30 seconds to evaluate the stability of the material under photothermal conditions (at a concentration of 50 μg mL^-1^) over four cycles.

***In vitro* ROS Production**

**Evaluation of •OH Generation by TMB and MB**

In order to identify the presence of hydroxyl radicals (•OH), the color reaction involved using 3,3′,5,5′- tetramethylbenzidine dihydrochloride (TMB) as a probe, which turns blue when oxidized by •OH. CuNTD were mixed with TMB and H_2_O_2_ in PBS solution. The UV-vis spectrophotometer was used to measure the variation in absorbance of the solution. Under identical circumstances, the specimen was distributed in PBS with varying levels of H_2_O_2_, and the impact of a 1064 nm laser was assessed. The presence of •OH in a PBS solution with H_2_O_2_ was identified by monitoring the decrease in absorption peak of methylene blue (MB) at 660 nm. CuNTD or Ti_3_C_2_ was added in MB aqueous solution containing H_2_O_2_ and laser radiation at different times, the absorbance at 664 nm was measured by a UV-vis spectrophotometer.

**POD-like Activity and Enzymatic Kinetic Experiments**

The Beer-Lambert Law was used to [calculate](javascript:;) the initial reaction rates (V_0_) of varied TMB and H_2_O_2_ concentrations based on the absorbance change of oxTMB formation. The Michaelis-Menten kinetics curve was obtained by fitting the mathematical equations of the relationship between the reaction rate and the concentration of TMB and H_2_O_2_. Km and Vmax were measured by Lineweaver-Burk diagram.

$A=\varepsilon bc$ (1)

$v_{0}=\frac{v_{\max}\bullet\left[S\right]}{K_{m}+\left[S\right]}$ (2)

$\frac{1}{v_{0}}=\frac{K_{m}}{v_{\max}}\bullet\frac{1}{S}+\frac{1}{v_{\max}}$ (3)

**Singlet Oxygen (^1^O_2_) Generation**

1,3-Diphenylisobenzofuran (DPBF) serves as a marker for singlet oxygen (^1^O_2_) and can undergo a reaction with ^1^O_2_ when exposed to a 1064 nm laser (1 W cm^-2^), leading to a reduction in DPBF absorption around 410 nm. DPBF was combined with a solution of Ti_3_C_2_ or CuNTD (50 μg mL^-1^) and exposed to a 1064 nm laser with a power density of 1.0 W cm^-2^ for varying durations up to 30 minutes, followed by recording of the spectra.

**Evaluation of ROS Generation by DHR**

The ROS production of CuNTD was verified by ROS fluorescence probe dihydrorhodamine (Dihydrorhodamine, DHR), and DHR can be oxidized by ROS to a fluorescent ionic state. CuNTD nanozymes were mixed with DHR and H_2_O_2_. After irradiation treatment, fluorescence intensity was measured by fluorescence detector at different time.

***In Vitro* GSH** **Depletion**

The UV-vis spectroscopy was used to track the reduction of GSH. 5,5'-Dithiobis-(2-nitrobenzoic acid) (DTNB) was utilized for the detection of GSH in the solution. DTNB can undergo a reaction with the sulfhydryl group on GSH to produce the TNB^2-^ anion, resulting in a bright yellow color at 412 nm. A solution of CuNTD was combined with solutions of DTNB and GSH, followed by exposure to a 1064 nm laser at 1 W cm^-2^ for 4 minutes. Subsequently, UV-vis spectroscopy was used to measure the absorbance of the resulting mixtures.

**Cytotoxicity Assay**

Cytotoxicity assessments on various samples were conducted using the cell-counting kit 8 (CCK-8) assay. 96-well plates were used to seed HUVEC and 4T1 cells. Following a 24-hour incubation period, the original medium was exchanged with a new medium that included either Ti_3_C_2_ or CuNTD at various concentrations (12.5, 25, 50, and 100 μg mL^-1^). Following a 4-hour incubation period, the wells containing 4T1 cells from various groups were exposed to a 1064 nm laser for 4 minutes. Each group underwent an additional 20-hour incubation period before being exposed to CCK-8 solution in the wells for 4 hours. Subsequently, the absorbance at 450 nm was measured using a plate reader.

**Intracellular ROS Generation Detection**

Intracellular ROS and •O_2_^-^ were detected using 2,7-dichlorodihydro-fluorescein diacetate (DCFH-DA) and dihydroethidium (DHE) as probes. After incubating 4T1 cells in culture dishes for 20 hours, a medium solution containing CuNTD (50 μg mL^-1^) was added and incubated for an additional 4 hours. Subsequently, the cells were subjected to a 1064 nm laser (1 W cm^-2^) for 4 minutes. Following this, the 4T1 cells were rinsed using PBS. To conduct the complete ROS assay, the cells were treated with DCFH-DA for a duration of 10 minutes and then visualized using CLSM. In the O_2_^-^ analysis, the cells were stained for 10 minutes before being detected using CLSM.

**Mitochondrial Integrity Assay**

4T1 cells were cultured in 24-well plates for adherent growth and incubated for 20 h. Subsequently, the cells were incubated with CuNTD nanozymes. After 4 h of incubation, the cells were exposed to a 1064 nm laser (1 W cm^-2^) for 4 min. The medium was removed and replaced with JC-1 staining solution according to the manufacturer's protocol. The cells were washed 3 times with PBS and imaged by CLSM.

**Live/Dead Cell Staining Assay**

The calcein-AM and propidium iodide were used to evaluate the live cells and dead cells, respectively. 4T1 cells were seeded in a 24-well plate with a density of 1.0 × 10^5^ cells per well and incubated with CuNTD nanozymes (50 μg mL^-1^). As for laser irradiation groups, all the treatments were like that above and irradiated by a 1064 nm laser for 5 min. After all the treatment, 4T1 cells were stained by calcein-AM and PI and then imaged by CLSM.

**Apoptosis Detection Assay**

The apoptosis-mediated cell death was quantitatively analyzed by a flow cytometer. The cultured cells were seeded in a six-well plate evenly and cultivated for 24 h. Subsequently, the 4T1 cells were incubated with CuNTD nanozymes at the same concentration (50 μg mL^-1^) for 4 h. The laser irradiation groups were illuminated by a 1064 nm laser (1 W cm^-2^, 4 min). Then, all the treated cells were [collect](javascript:;)ed and stained by Annexin V-APC/PI and then analyzed with a flow cytometer.

**Intracellular GSH Content**

4T1 tumor cells were seeded into a 24-well plate to allow attachment. Then, these 4T1 tumor cells were incubated for 20 h and treated with CuNTD (50 μg mL^-1^). After 4 h, these cells were collected and intracellular GSH contents were detected by an GSH and GSSG assay kit. In addition, ThiolTracker™ Violet dye is an intracellular thiol probe for monitoring intracellular GSH consumption. After the same treatment as above, the cells were stained at 37℃ for 30 minutes and the images were obtained under a fluorescence microscope. Subsequently, the fluorescence intensity was [evaluate](javascript:;)d by with a flow cytometer.

**Measurement of [Extracellular](javascript:;) ATP Level**

To measure extracellular ATP release levels, 4T1 cells were seeded in a 6-well plate at a density of 2×10^5^ cells per well and cultured for 20 h. Then, the medium was then replaced with fresh medium containing control and CuNTD for 4 h. The laser irradiation groups were illuminated by a 1064 nm laser (1 W cm^-2^, 4 min). The supernatant was collected 2 h after irradiation and used for measurement of the intracellular ATP level via an ATP testing kit.

***In Vitro* Detection of ICD Markers**

The expression of CRT and HMGB1 in 4T1 cancer cells was detected by immunofluorescence assay. 4T1 cells were first treated with CuNTD, then washed twice with frozen PBS, fixed with 4% paraformaldehyde (PFA) for 30min, and permeated with 0.2% PBST at room temperature for 30min. For CRT, after blocking with 5% FBS, staining with mouse anti-CRT (1:2000) primary antibody at 4°C overnight, followed by staining with AF488-coupled goat anti-mouse secondary antibody (1:2000 dilution). For HMGB1, after blocking with 5% fetal bovine serum, rabbit anti-HMGB1 (1:4000) primary antibody was stained overnight at 4℃, and then AF647-coupled goat anti-rabbit secondary antibody (1:4000 dilution) was stained. After the unattached antibodies were washed away, the nucleus was stained with Hoechst-33342 for 5min and imaged under CLSM.

***In Vivo* Immune Evaluations**

This study was based on Balb/c mice with double tumors, which were treated in the same way as the single tumors described above. On the 14th day after treatment, three mice in each group were randomly killed and spleen and lymph nodes were collected. To show the infiltration of immune cells, the harvested organs were analyzed by flow cytometry for population analysis of T cells and DCs. Spleen and lymph nodes were subjected to DC maturation with anti-CD80 and anti-CD86 antibodies. Spleen T cells were stained with anti-CD3^+^CD4^+^ and anti-CD3^+^CD8^+^ antibodies. On day 28, mouse lung tissue was collected for metastasis detection. The survival period of the mice was measured by 60 days of records.

Single-cell suspension of the spleen and lymph nodes was prepared for the above flow cytometry analysis. Firstly, the spleen and lymph nodes were ground with a grinding rod, filtered with a 200-mesh sieve, and the suspension was rinsed with PBS. Then, disperse cells were stained with fluorescence-conjugated antibodies described above. After labeling, the cells were quantitatively analyzed by flow cytometry.

***In Vitro* Hemolysis Assay.**

50 μL of fresh blood from BALB/c mice was added into 1.95 mL of CuNTD nanozymes saline solution with different concentrations. Simultaneously, as a control, 50 μL of fresh blood was added into 1.95 mL of deionized water. After 12 h of incubation, all samples were centrifuged, and the absorbance of the supernatant was measured using a UV-vis spectrophotometer.

**Supplementary Figures**


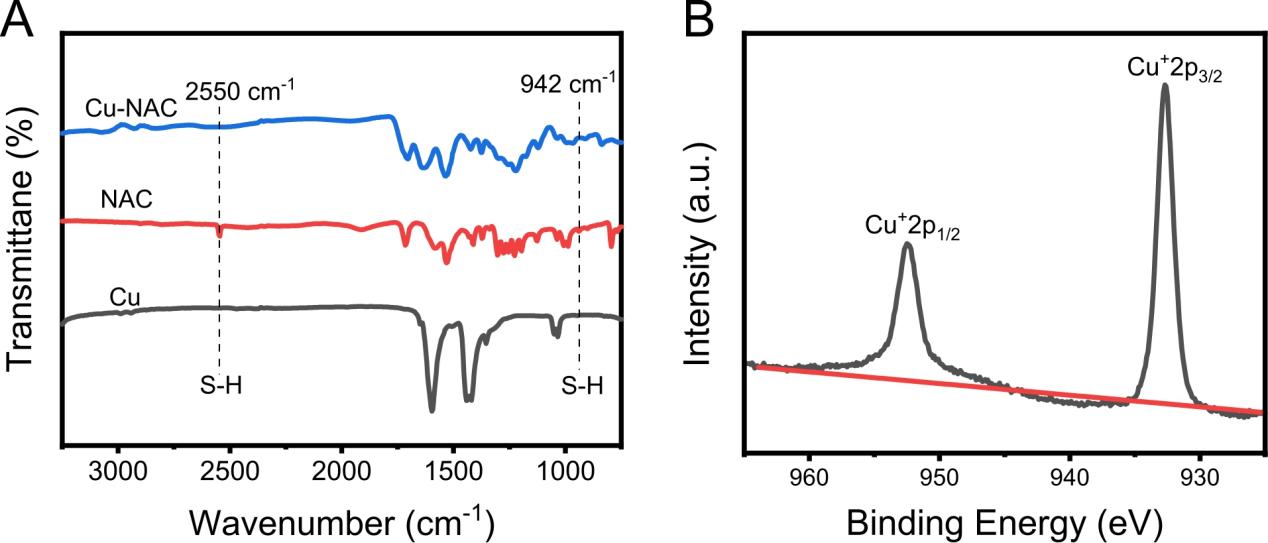


**Figure S1.** (A) FTIR spectra of Cu-NAC complex, NAC, Copper acetate. (B) XPS spectra of Cu 2p in Cu-NAC complex.


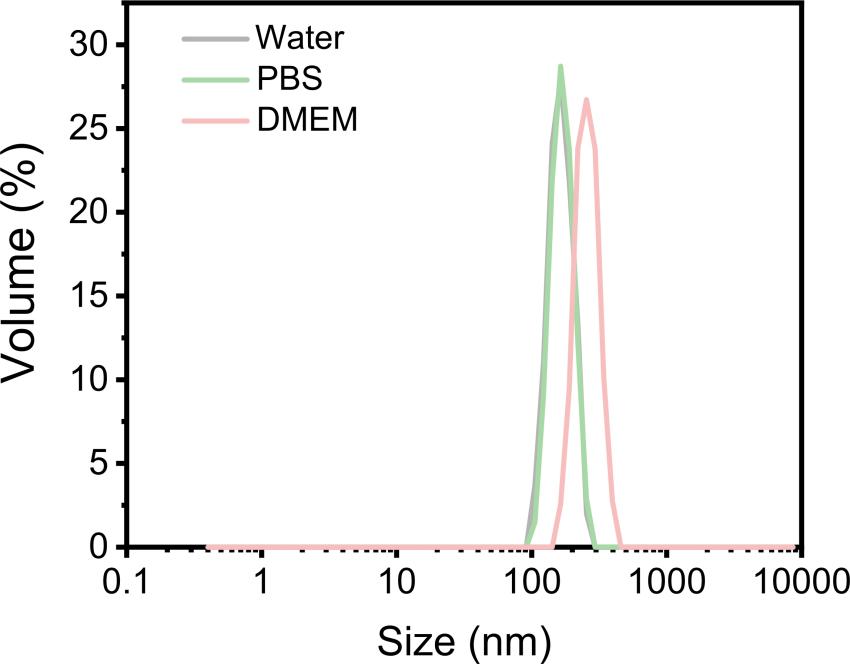


**Figure S2.** The hydrodynamic diameter of CuNTD dispersed in water, PBS and DMEM tested by DLS.


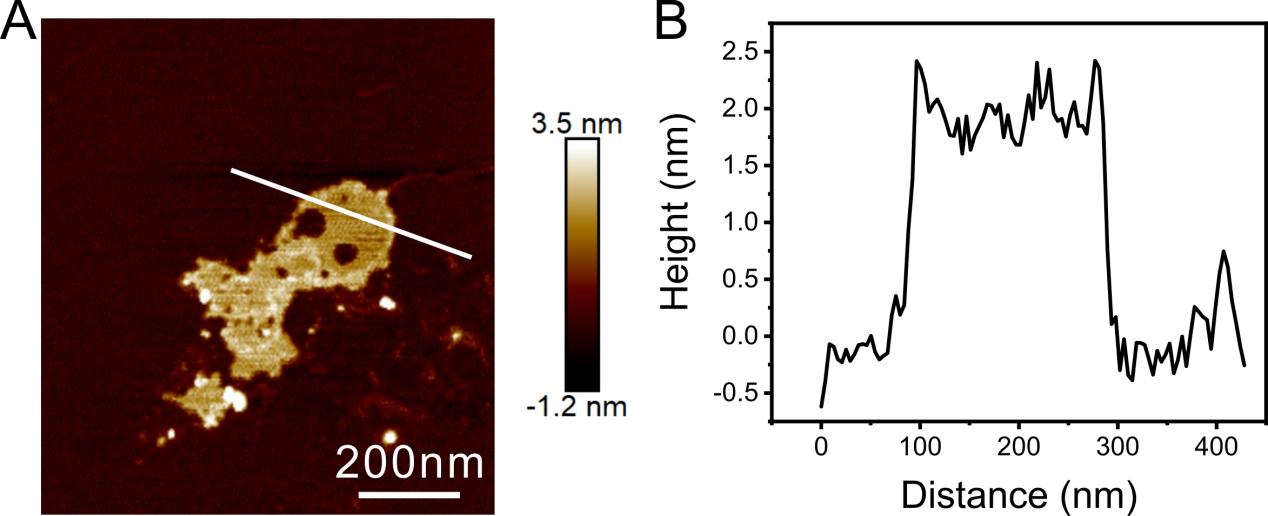


**Figure S3.** (A) CuNTD was measured by atomic force microscopy (AFM). (B) Thickness of CuNTD measured from AFM image.


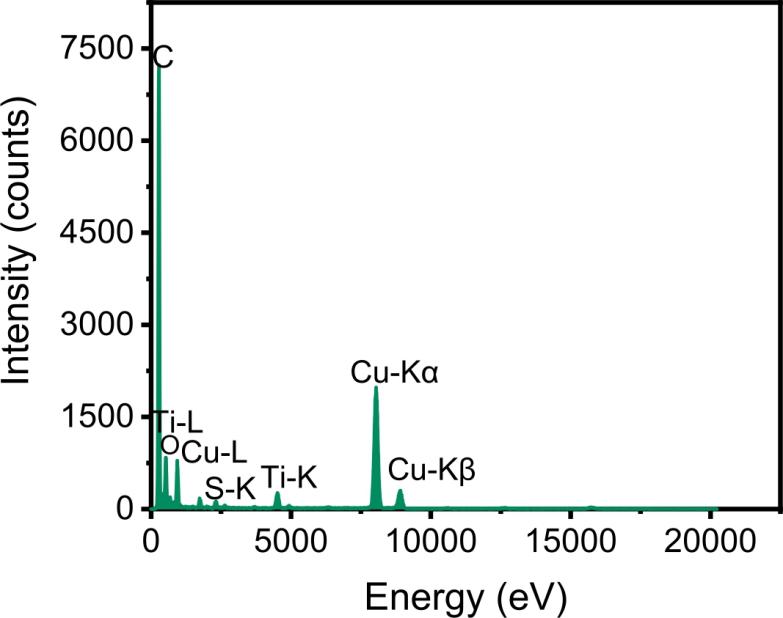


**Figure S4.** EDS mapping images of the CuNTD.


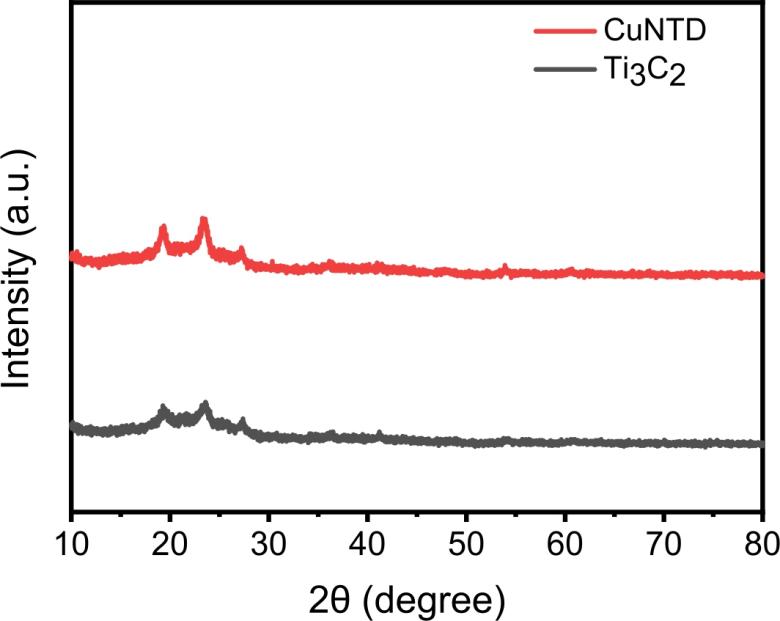


**Figure S5.** XRD spectra of Ti_3_C_2_ and CuNTD.


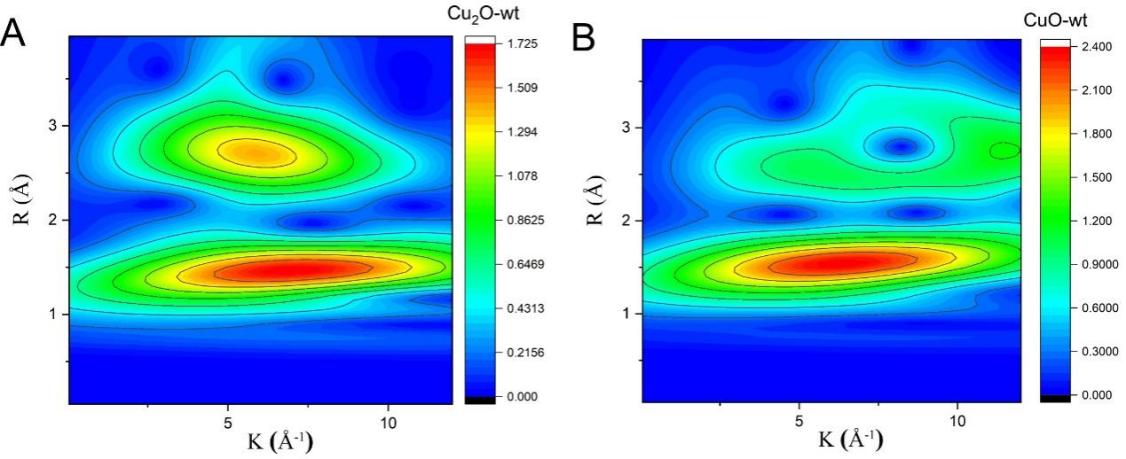


**Figure S6.** (A, B) Wavelet transform of Cu_2_O and CuO.


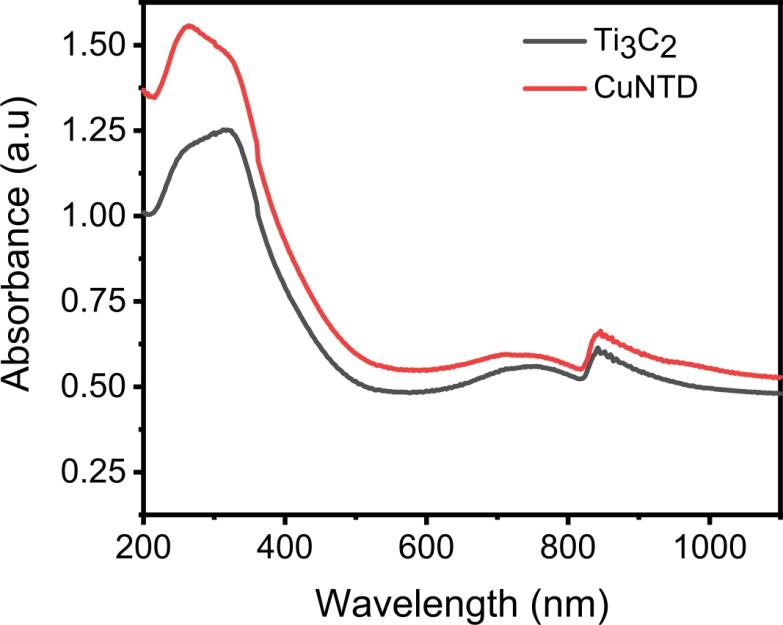


**Figure S7.** UV-vis-NIR absorption spectra of Ti_3_C_2_ and CuNTD (50 μg mL^-1^).


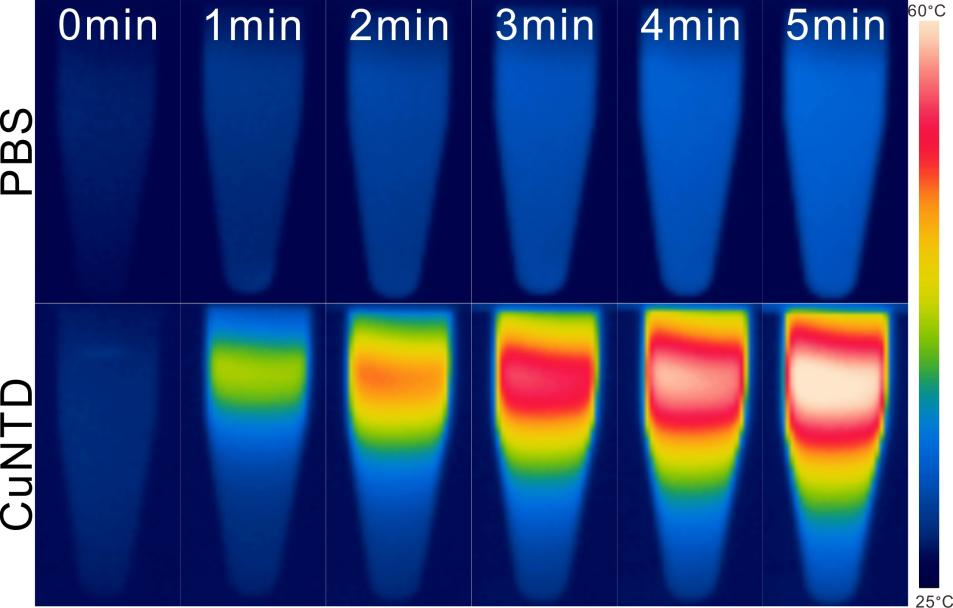


**Figure S8.** Infrared thermal images of the CuNTD aqueous solution (100 μg mL^-1^) and PBS under irradiation by a 1064 nm laser with a power density of 1W cm^-2^ for 5 min.


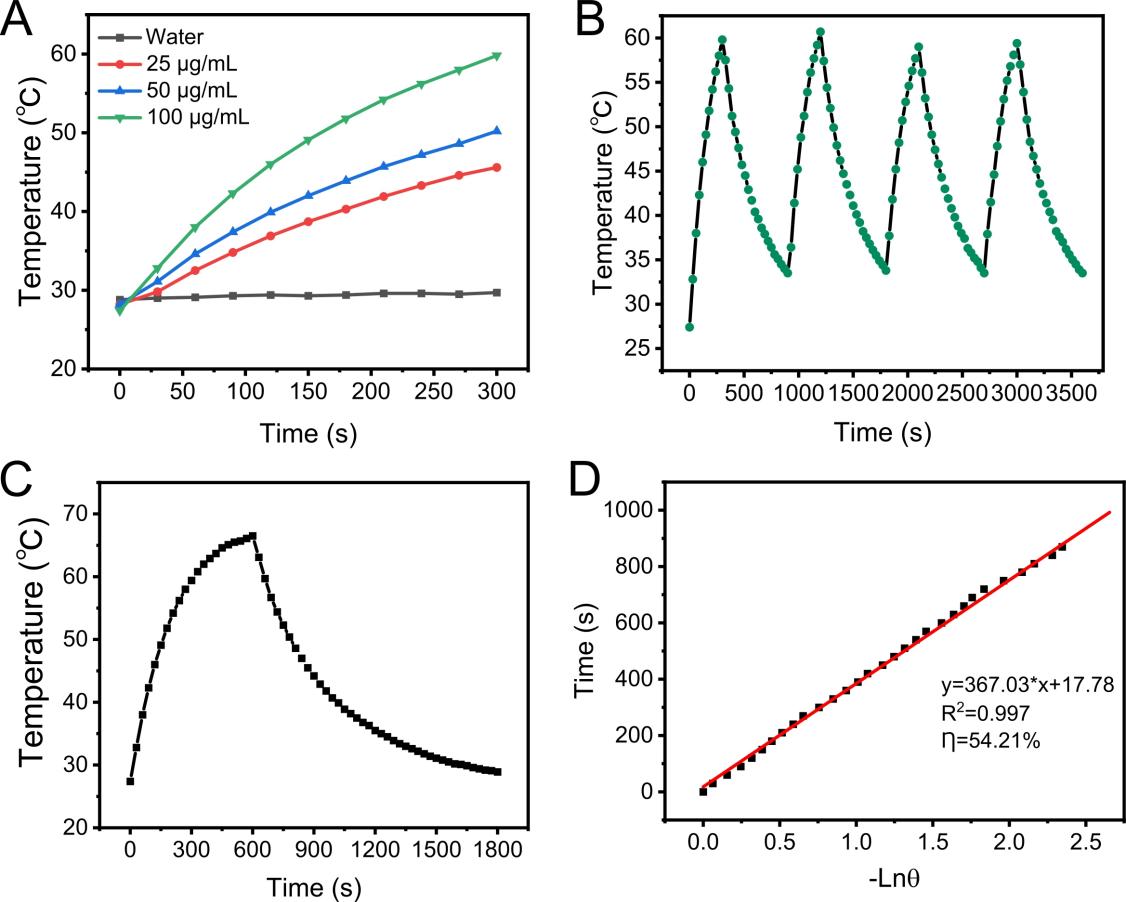


**Figure S9.** (A) Temperature variation curves for CuNTD aqueous solution with different concentrations under 1064 nm laser irradiation at 1 W cm^-2^. (B) Recycling-heating profiles of CuNTD solution under 1064 nm laser irradiation at 1 W cm^-2^ for four laser on/off cycles. (C) The photothermal effect of CuNTD aqueous solution irradiated with a 1064 nm laser (1 W cm^-2^), in which the irradiation lasted for 600 s, and then the laser was shut off. (D) Linear time data versus -Lnθ obtained from the cooling period as shown in (C).


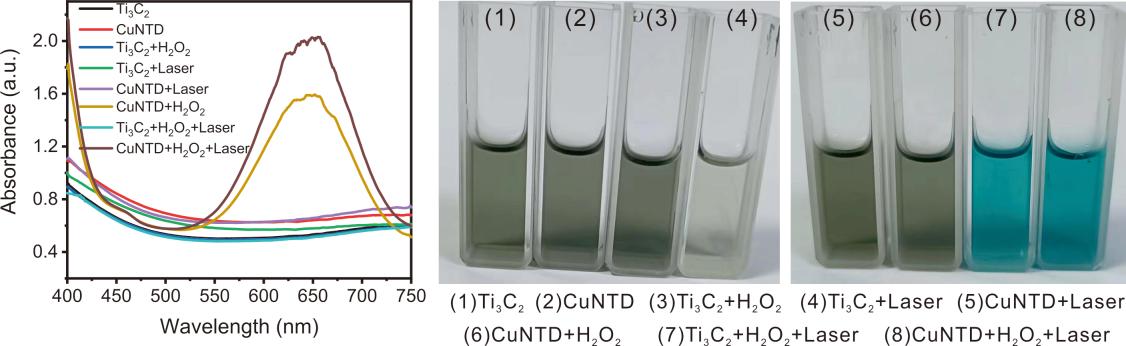


**Figure S10.** UV-vis absorption spectra of the catalyzed oxidation of TMB (oxTMB) under different conditions. The right are the corresponding visual color changes.


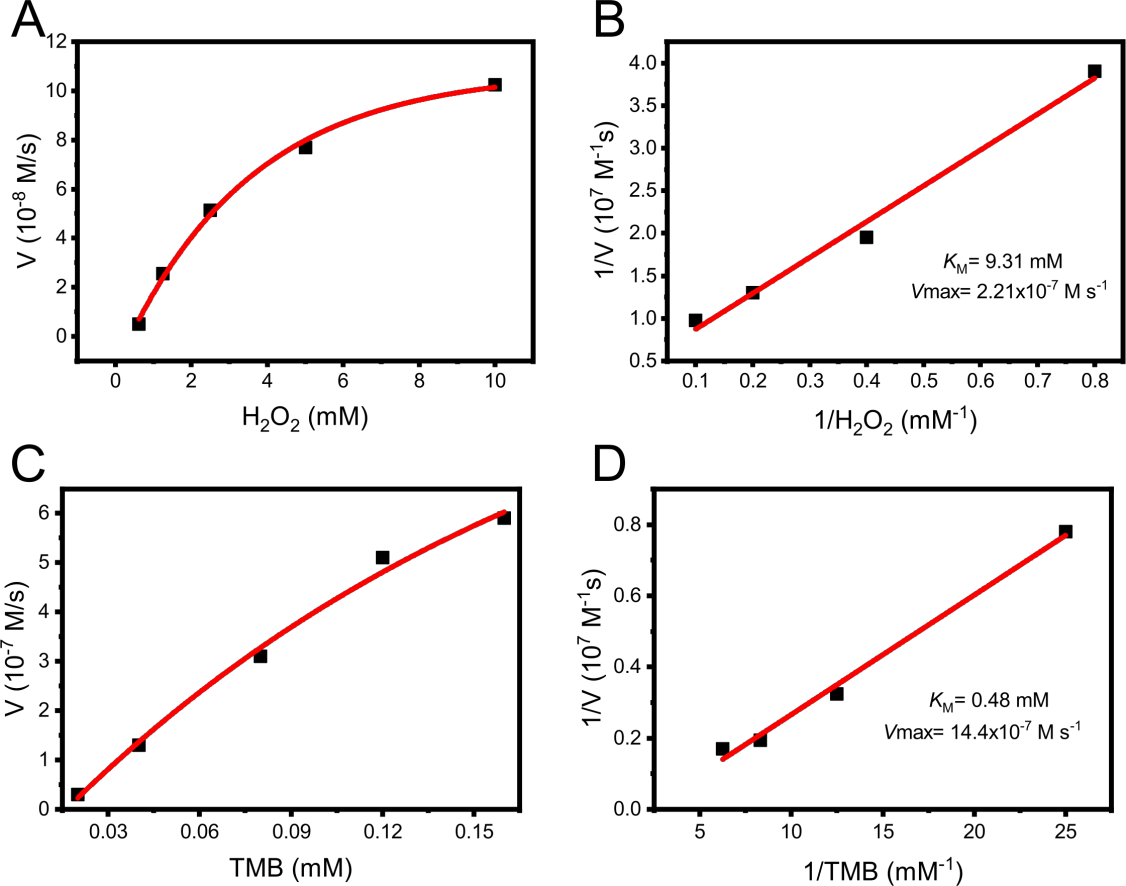


**Figure S11.** Michaelis–Menten kinetic analysis (A, C) and (B, D) Lineweaver–Burk plotting of POD-like activity.


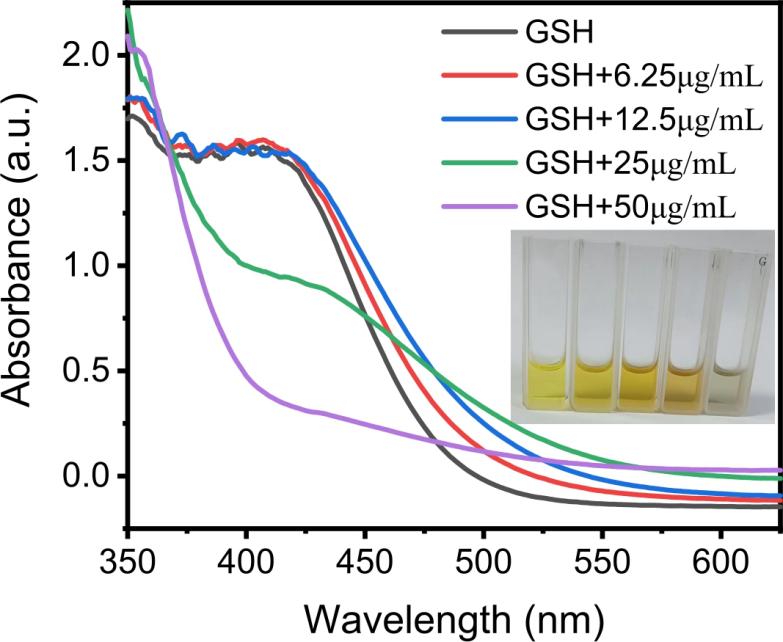


**Figure S12.** Concentration-dependent GSH depletion by CuNTD. Insert shows the corresponding photograph of solution.


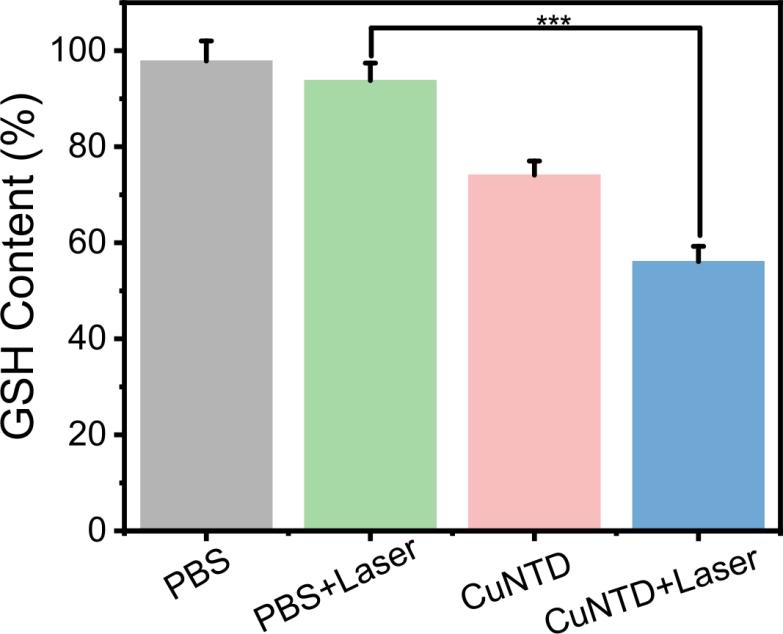


**Figure S13.** Intracellular GSH content after various treatment (n=3). Statistical significance was calculated using two-tailed Student’s t test: *p < 0.05, **p < 0.01, and ***p < 0.001.


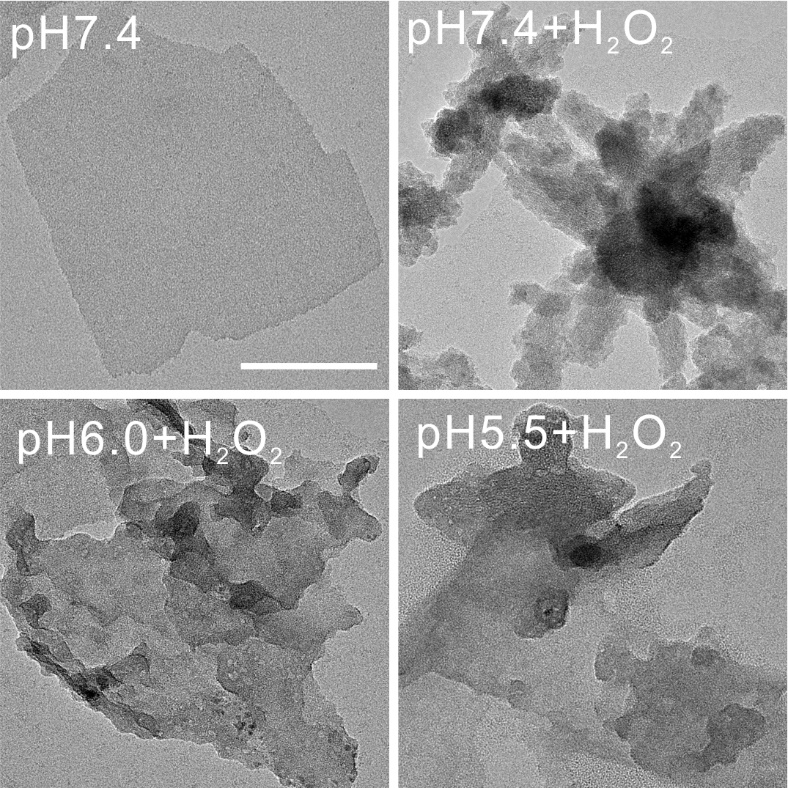


**Figure S14.** TEM images of CuNTD after different treatments for 24 h. The scale bar is 100 nm.


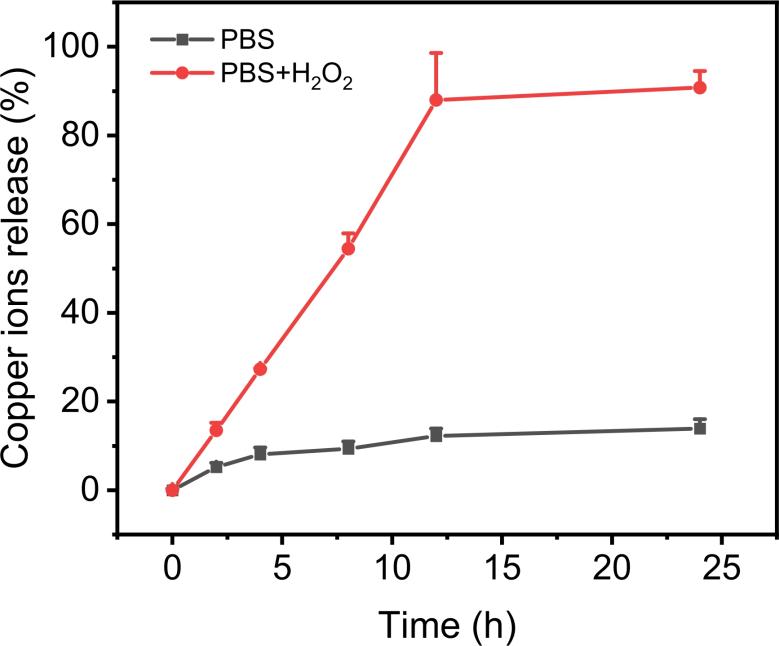


**Figure S15.** Copper ions release in the presence of H_2_O_2_ and PBS at different time points.


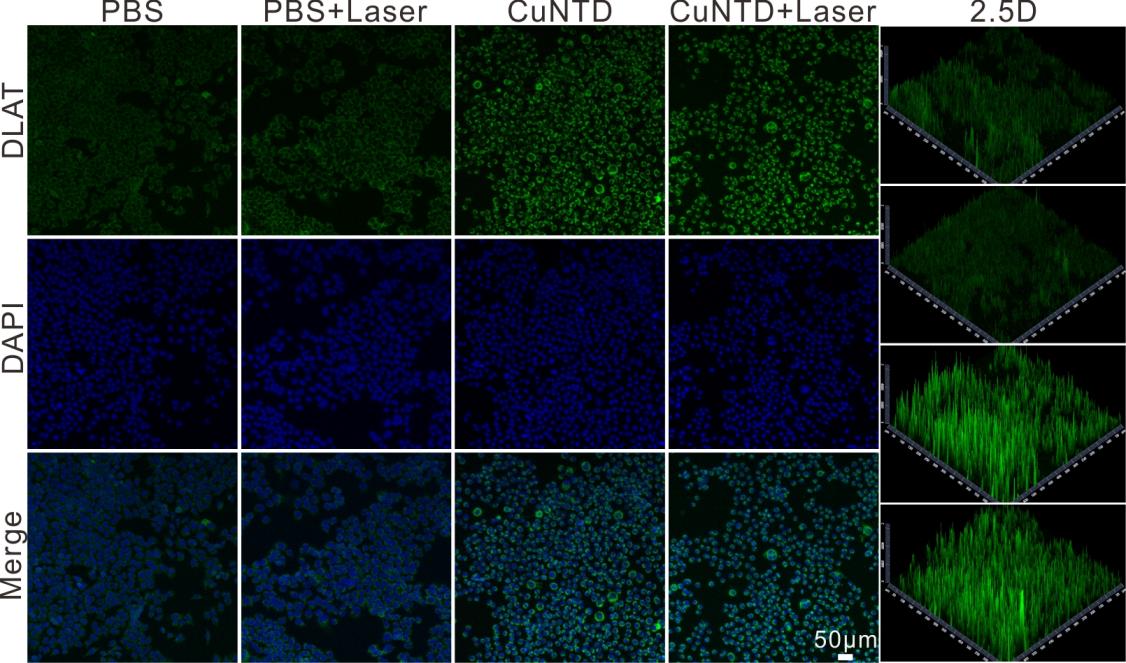


**Figure S16.** CLSM images of DLAT expression of 4T1 cells after different treatments.


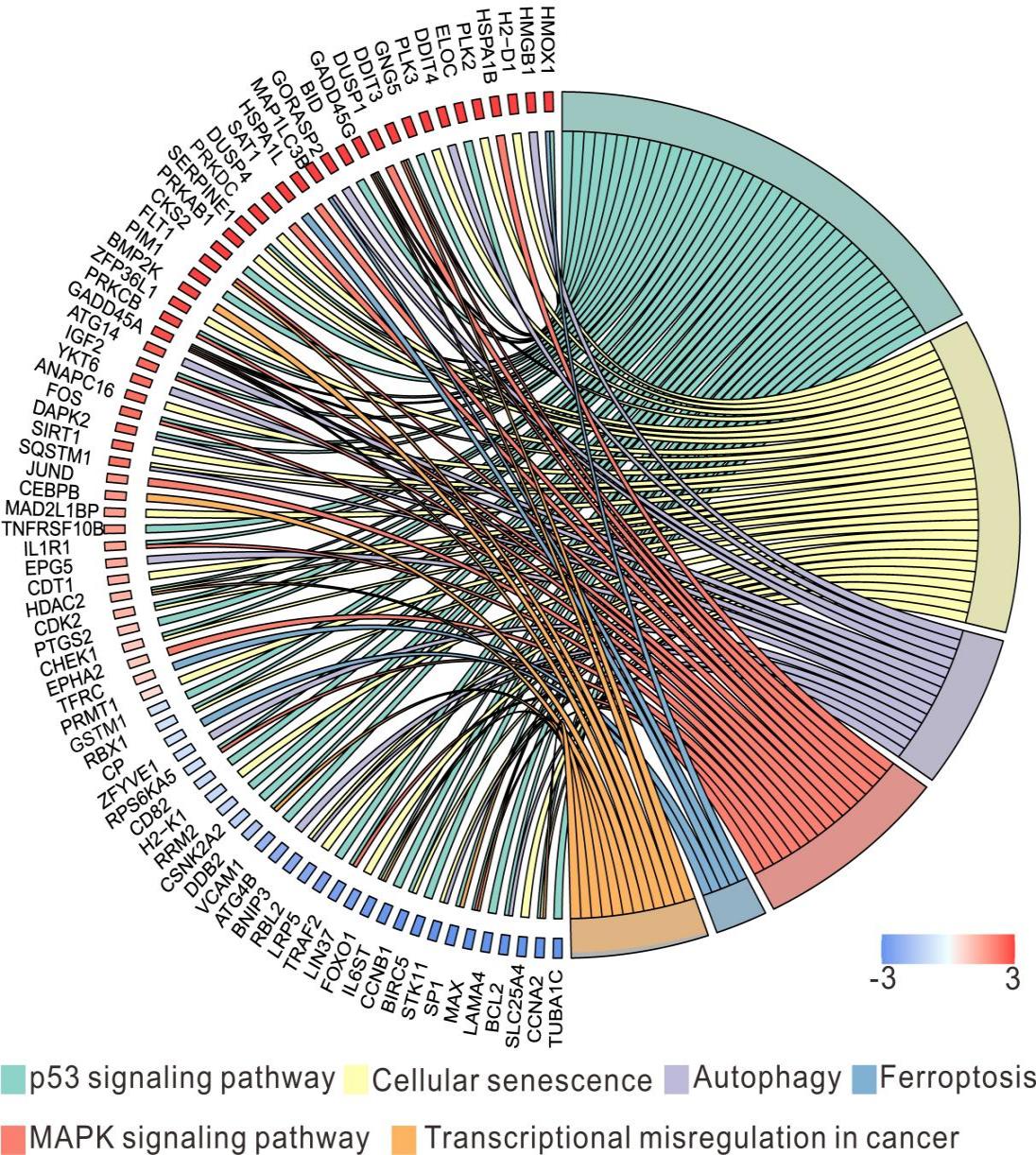


**Figure S17.** KEGG pathway enrichment analysis upon treatment with PBS and CuNTD.


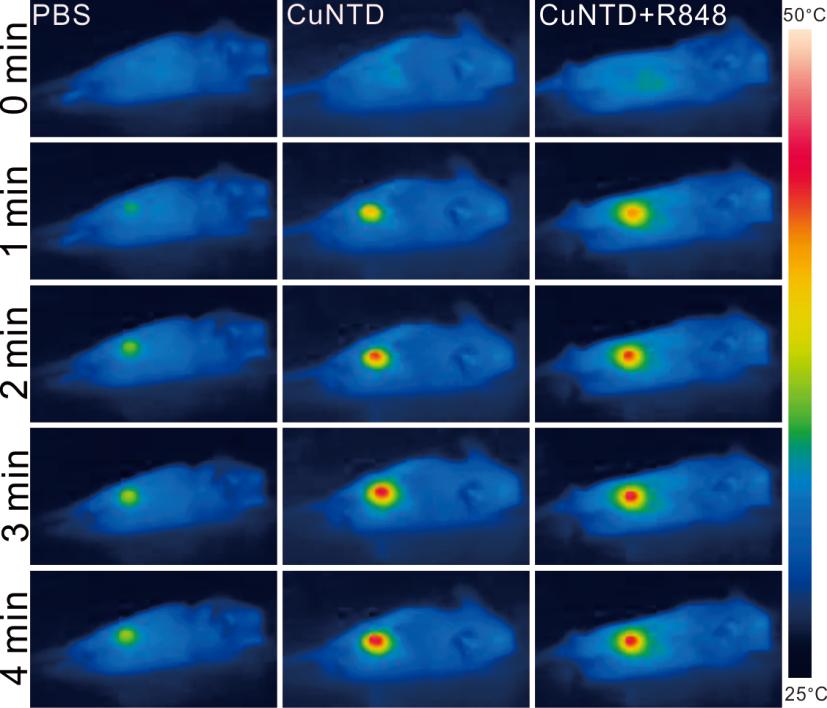


**Figure S18.** Infrared thermal images of 4T1 tumor-bearing mice of the tumors after [intratumor injection](javascript:;) of CuNTD under laser irradiation (1064 nm, 0.5 W cm^-2^) for 4 min.


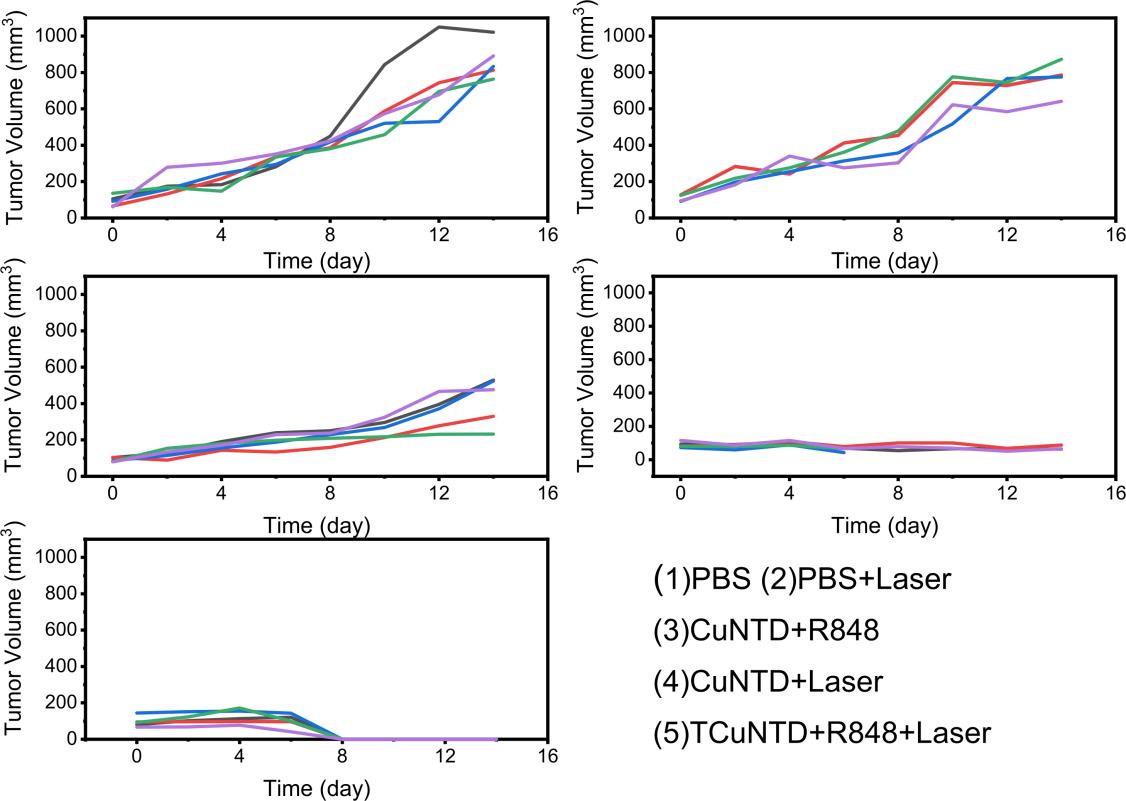


**Figure S19.** Individual tumor growth curves of 4T1-tumor-bearing mice after different 14-day treatments.


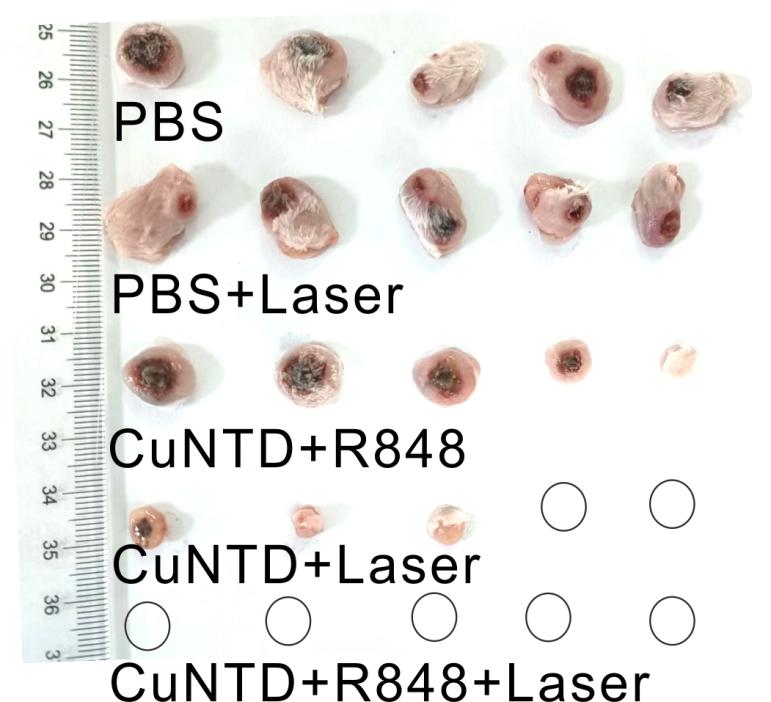


**Figure S20.** Images of isolated tumors in different groups on day 14.


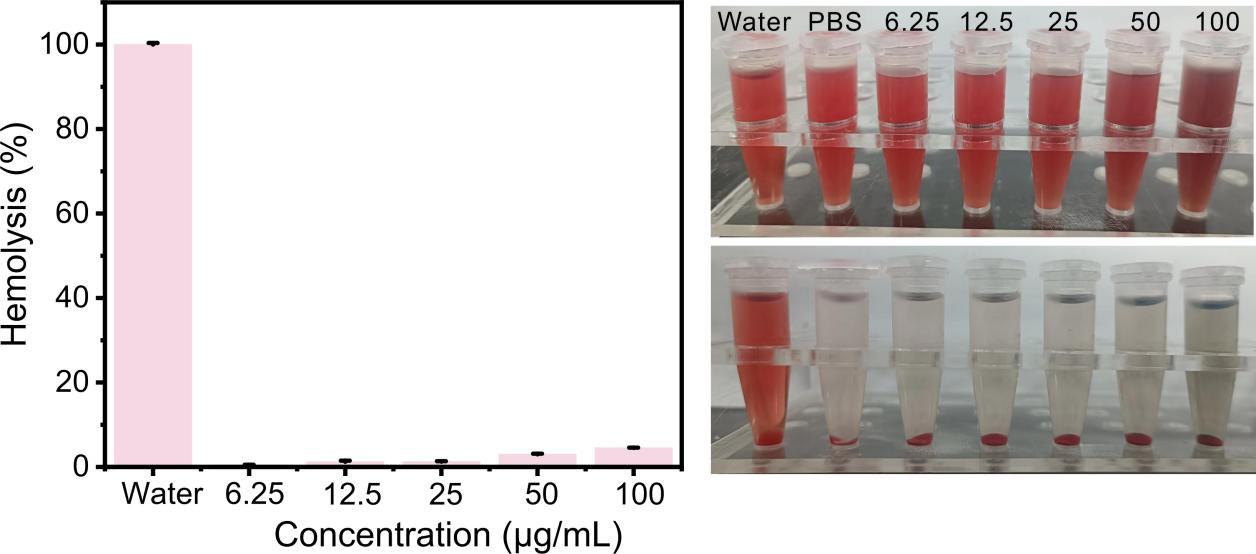


**Figure S21.** Hemolysis assay using mouse erythrocytes when incubated with CuNTD of various concentrations (n=3).


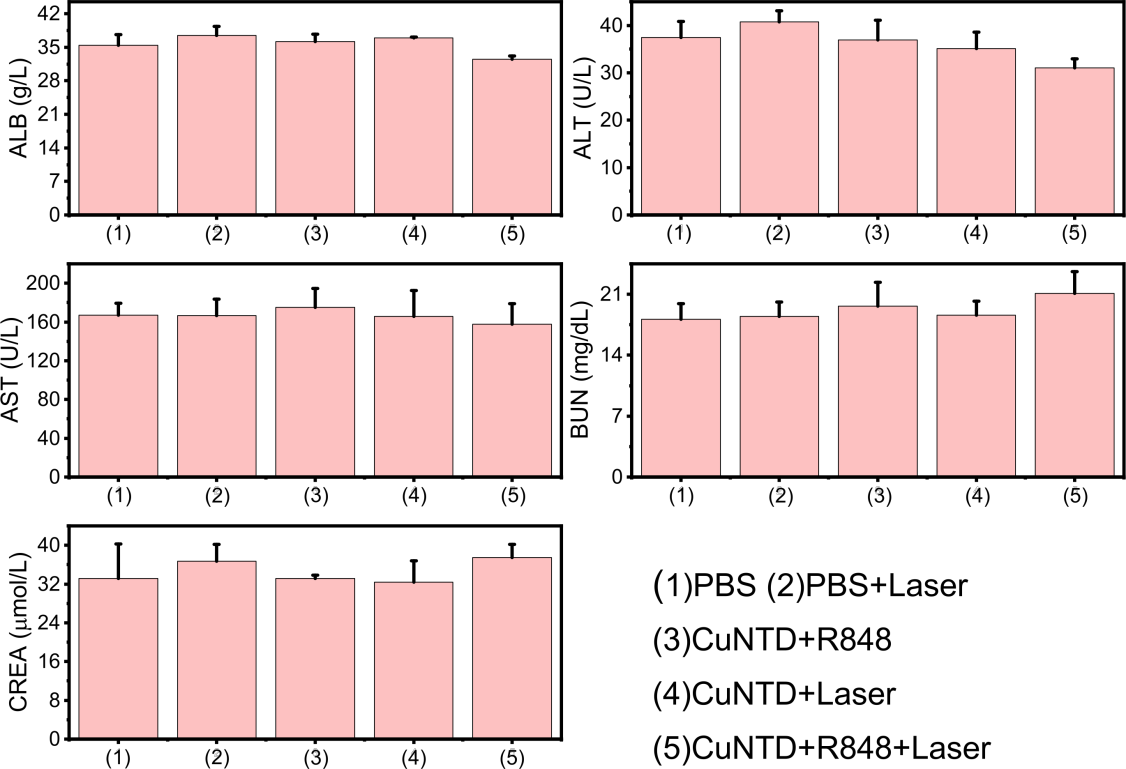


**Figure S22.** The biochemical parameters of liver and kidney after treated with different treatments (n=5).


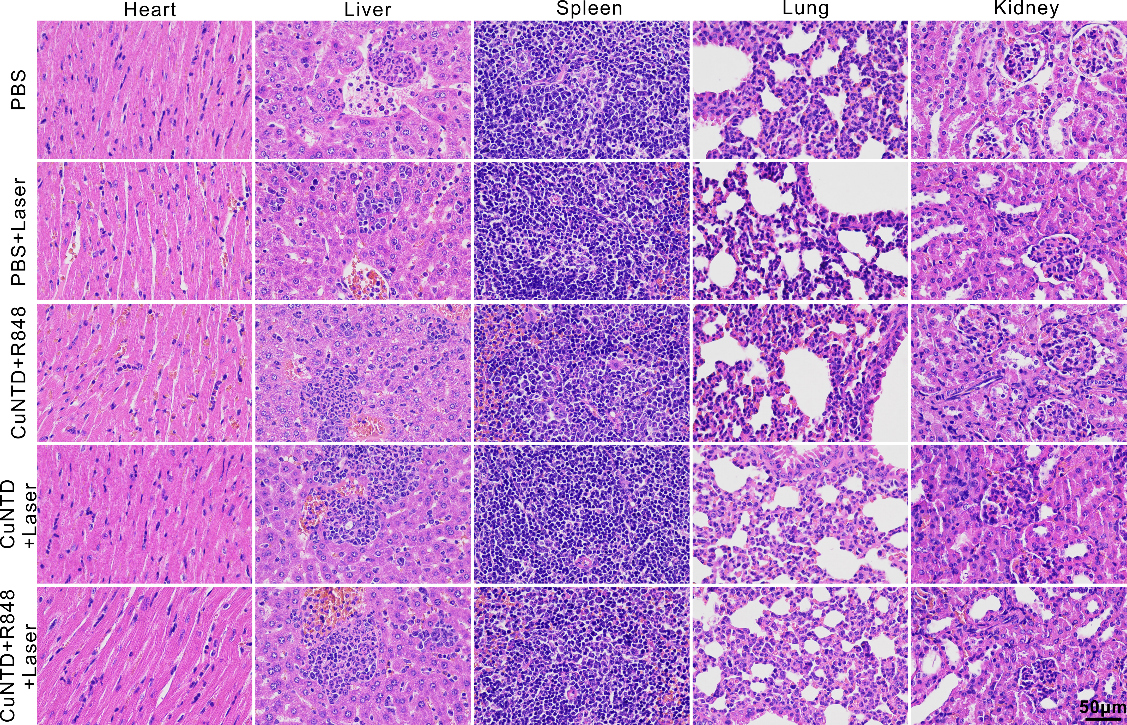


**Figure S23.** H&E staining of major organs after treated with different treatments.


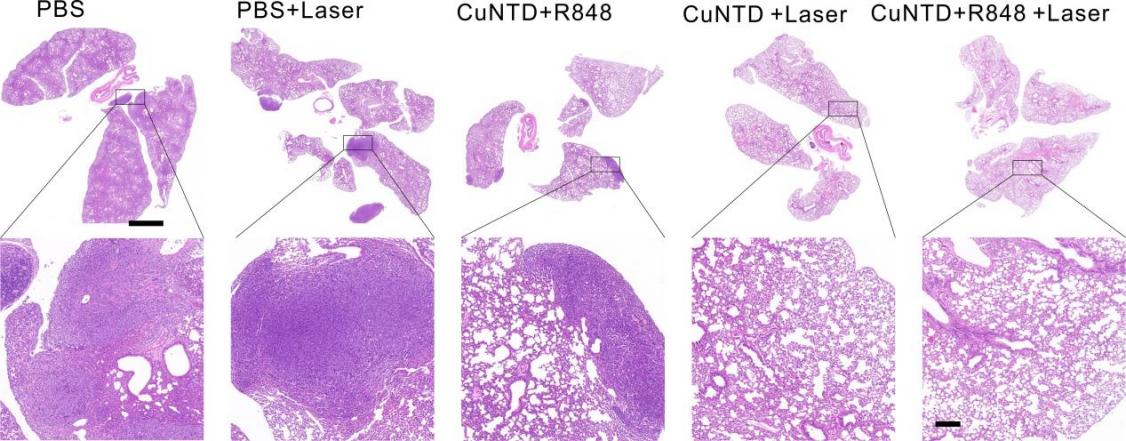


**Figure S24.** H&E-stained images of lung tissues in different treatment groups. The scale bar is 2 mm and 0. 2 mm.

**Supplementary Table**

**Table S1.** EXAFS fitting parameters at the Cu K-edge various samples.

| Sample | Shell | CN | R(Å) | σ^2^ | ΔE_0_ | R factor |
| --- | --- | --- | --- | --- | --- | --- |
| Cu foil | Cu-Cu | 12 | 2.54±0.01 | 0.0087 | 4.7±0.5 | 0.0030 |
| Cu AcAc | Cu-O | 4.6±0.3 | 1.95±0.01 | 0.0070 | -8.2±1.8 0.0083 | |
|  | Cu-Cu | 1.0±0.3 | 2.64±0.02 | 0.0093 |  |  |
| CuNTD | Cu-S | 2.7±0.1 | 2.24±0.01 | 0.0077 | -1.0±1.3 | 0.0153 |

C. N.: coordination numbers; R: bond distance; σ^2^: Debye-Waller factors; ΔE_0_: the inner potential correction. R-factor: goodness of fit. *The experimental EXAFS fit by fixing C. N. as the known crystallographic value.
